# Quantifying UK coastal flood exposure under future sea-level rise to 2300

**DOI:** 10.1038/s41467-026-74982-1

**Published:** 2026-07-21

**Authors:** M. D. Palmer, J. Savage, P. D. Bates, J. Neal

**Affiliations:** 1 Met Office Hadley Centre, Exeter, UK; 2 School of Earth Sciences, University of Bristol, Bristol, UK; 3 Fathom, Bristol, UK; 4 School of Geographical Sciences, University of Bristol, Bristol, UK

**Keywords:** Projection and prediction, Climate-change impacts

## Abstract

The latest Intergovernmental Panel on Climate Change assessment report highlighted the potential for more than 15 m of global sea-level rise by 2300. In this study, we explore the implications for UK coastal flood exposure by combining national-scale flood modelling with physically-based storylines of UK sea-level rise. By 2100 all storylines show broadly similar results with at least an additional 0.5 million people exposed to the 1-in-200 year undefended flood extent, which represents a 25% increase compared to present day. Under the most pessimistic storyline by 2300 involving significant ice-sheet instability, an additional 13 million people could be exposed to the 1-in-200 year undefended flood event. This would imply the potential need for large-scale movement of populations and settlements away from the coast in the coming centuries. Given current global emissions pledges, exposure increases by 1.7 million people by 2300, however up to 1 million could be avoided if Paris Agreement targets for greenhouse gas emissions are met.

## Abstract (web summary)

1 million extra people will be exposed to coastal flooding in the UK by 2100 due to sea-level rise. Under a plausible worst-case scenario this rises to 13 million by 2300, but most of this risk can be avoided through pursuing Net Zero emissions policies.

## Introduction

Sea-level rise poses an existential threat to coastal communities and ecosystems around the world^1^. Global mean sea level (GMSL) increased by about 20 ± 5 cm over the period 1901–2018, with a marked acceleration since the 1960s^2^. Since the 1990s, the net contribution from ice sheet mass loss has increased by a factor of four, and these huge ice reservoirs remain the primary threat for sea-level rise in the coming centuries^2,3^. The UK is an ideal natural laboratory in which to examine the implications of these possible changes, given the large coastal flood exposure and complex interaction of different sea-level rise drivers for this location. While the observed increase in UK-average sea level of 18 ± 3 cm over the period 1900–2022 is in good agreement with GMSL, there are important spatial variations in the UK rate of rise associated with Glacial Isostatic Adjustment (GIA)^4^. In England alone, a recent study suggests that there are currently 544,000 residential and 72,000 non-residential properties in the coastal floodplain, an amount equivalent to 2.5% of the total national building stock^5^. Even when flood defences are accounted for, approximately 10% of these coastal floodplain properties are exposed to events with a return period of less than 75 years. This could increase substantially under sea-level rise scenarios, and this has the potential to significantly reshape the geography of the UK coast.

Previous studies have highlighted the consequences of climate policy decisions in the next few decades for determining sea-level rise outcomes for centuries and millennia to come, illustrating the importance of early emissions reductions and raising questions about intergenerational justice^2,6,7^. The IPCC Sixth Assessment Report of Working Group I (IPCC AR6) stated that by 2300, GMSL will increase by 0.3–3.1 m under low emissions (SSP1-2.6) and 1.7–6.8 m under high emissions (SSP5-8.5), increasing up to 16 m for SSP5-8.5 if uncertain ice sheet instability processes are included^2,8^. This wide range of potential outcomes has motivated recent studies to pursue a storyline approach, where a small number of singular and physically-consistent trajectories of future sea-level rise are generated that span the overall uncertainty space^9,10^.

The current national UK sea-level projections do not include the possibility of accelerated sea-level rise associated with uncertain ice sheet instability processes^11,12^, and current UK guidance on a “credible maximum (H++)” sea-level rise by 2100 is 1.9 m^13^ (www.gov.uk/guidance/flood-and-coastal-risk-projects-schemes-and-strategies-climate-change-allowances), yet sea-level rise in London could reach 5 m by 2150 and more than 15 m by 2300, based on outcomes highlighted in IPCC AR6^2,8,10^. Therefore, the goal of the present study is to provide a comprehensive analysis of the implications of IPCC AR6 sea-level projections out to 2300 for UK coastal flooding and coastline retreat. While coastal flood hazard is also subject to changes in storm surges, waves and tides, these are secondary factors compared to the century timescale threat from sea-level rise^14^ and therefore neglected from this study.

Current estimates of coastal flooding produced by the UK Government and others often use so-called ‘bathtub models’ to predict inundation. These take the extreme water level at the coast and project it inland, with the assumption that any land below this elevation is inundated even if, in practice, this is physically impossible due to dynamical effects or a lack of hydraulic connectivity^15^. Recent high-profile global studies looking at sea-level rise impacts^16–20^ also invariably use bathtub methods. Instead, to realistically model coastal inundation, a numerical hydrodynamic model is needed that properly represents the physics and time dynamics of shallow water flow. Such models have previously only existed for individual coastal segments or river reaches, but recently the availability of better data, new numerical schemes and High Performance Computing has allowed hydrodynamic approaches to be applied to simulate flooding at national to global scales^21–24^.

Our analysis is based on model simulations without flood defences for several reasons. Firstly, it allows us to identify: (i) new populations that could become exposed to flooding in the absence or failure of flood defences; (ii) infrastructure or land that could become exposed to floods, which could help inform decisions on whether areas require future investment in flood protection infrastructure; and (iii) locations that could be repurposed for relocated communities to inhabit. Secondly, there are no datasets that project flood defence standards out to 2300, and the level of investment in defences would also likely vary depending on the climate scenario followed. Lastly, if we assume defences remain at their current standards of protection, we would learn very little about changing flood risk except in undefended and rural areas. Therefore, we feel that the undefended scenario provides a more complete picture of potential exposure to floods both in the present day and the future. We work with the present day population as a baseline because currently available UK future projections^25^ are: (i) ‘blind’ to flood risk and therefore allow expansion of population into areas which, in reality, would never be developed; (ii) only available at 1 km resolution which is insufficiently granular for a localised problem such as flooding; and (iii) only exist out to 2100 as beyond this uncertainties are simply too great. It should be noted, however, that the population is not static and future work should incorporate these effects once appropriate projections become available.

This study uses a recently published set of physically-consistent storylines of UK future sea-level rise that span the full range of potential outcomes to 2300 across the low emissions (SSP1-2.6) and high emissions (SSP5-8.5) scenarios, including ice sheet collapse, highlighted in IPCC AR6^3,10^. While we acknowledge that high emissions futures are considered unlikely^26^, the sea-level rise outcomes associated with simulations run under these scenarios are important for a more complete understanding of the total risk landscape and for risk-averse decision contexts^27,28^. These are then used within the 1 arcsecond (25 m) spatial resolution whole UK hydrodynamic model of Bates et al.^23^ to simulate the area at risk of flooding during storm events, given the different sea-level rise scenarios. This whole UK model is unique in being able to simulate coastal inundation from any plausible boundary conditions with fine spatial resolution and high skill (see “Methods” for more details). The storylines are based on the same underlying projection machinery as the UKCP18 sea-level projections^11,29^ and are therefore consistent with that information, which forms the basis of current UK planning. The results presented here are based on the five storylines of future UK sea-level rise from Palmer et al.^10^ with a focus on two for the flood modelling results. Storyline B is used as a baseline scenario and is broadly consistent with current international greenhouse gas emissions pledges. Storyline H2 is used as a “reasonable worst case scenario” under high greenhouse gas emissions and includes representation of poorly-understood ice sheet instability processes (see Supplementary Note S1). While both H1 and H2 could be used as a reasonable worst-case scenario, we focus on H2 because it corresponds to the Low-Likelihood High-Impact storyline that was given prominence in the IPCC AR6 Summary for Policymakers and more completely illustrates the risk that could be avoided through effective global action to reduce greenhouse gas emissions^28^. In this work, we characterise the coastal flood impacts at 2100, 2200 and 2300 associated with the sea-level rise storylines given the present-day population for the UK as a whole and for the UK capital cities of Belfast, Cardiff, London and Edinburgh. Simulations are based on the 1-in-200-year return period extreme coastal water level event from the UK Environment Agency coastal flood boundary condition dataset (see “Methods”) since this is the typical design standard of UK coastal flood defences in urban areas.

## Results

### Sea-level rise storylines and spatial patterns

**Fig. 1:**
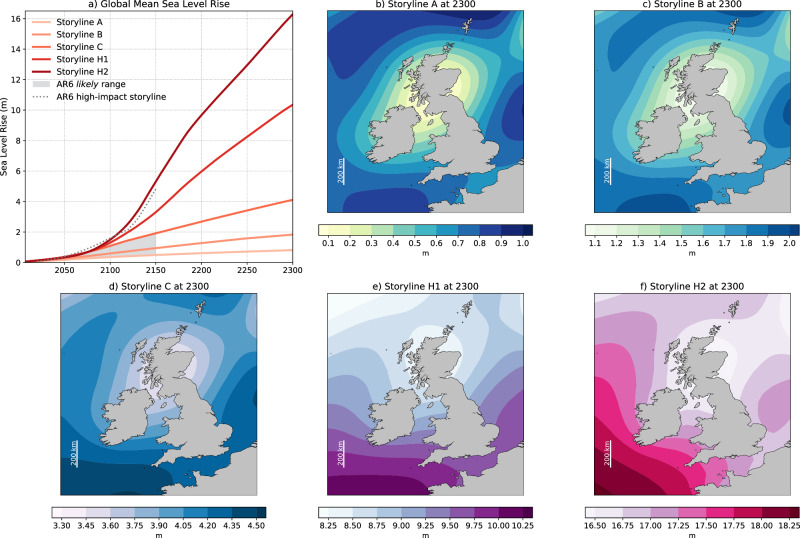
sea-level rise storylines and 2300 spatial patterns. **a** Time series of global mean sea-level (GMSL) rise for the five storylines presented in Palmer et al.^10^ as indicated in the figure legend. The grey-shaded region shows the overall IPCC AR6 *likely* range (i.e., the central two-thirds probability distribution) across the SSP1-2.6 and SSP5-8.5 greenhouse gas emissions scenarios to 2150. The grey dotted line shows the IPCC AR6 low-likelihood high-impact storyline presented in the Working Group I Summary for Policymakers^3^. **b**–**f** The spatial pattern of relative sea-level rise at 2300 associated with each storyline. All sea-level rise projections are expressed relative to the 1986–2005 average.

The storylines used in this study represent a broad range of outcomes for global mean sea-level (GMSL) rise from less than 1 m to more than 16 m by 2300 (Fig. 1a, Table 1). Story A, B and C show a near-linear rise of GMSL and span the overall range of the *likely* projections from IPCC AR6 across the SSP1-2.6 and SSP5-8.5 emissions scenarios. Storylines H1 and H2 show a marked acceleration in GMSL rise between 2050 and 2150 associated with ice sheet instabilities and correspondingly much larger rates and magnitudes of sea-level rise over the coming centuries. The model simulations underlying the storylines are based on assumptions of reducing greenhouse gas emissions^30^ from the 2020 s (Storyline A), around 2040 (Storyline B) or from 2150 (Storylines C, H1, H2) with a subsequent decrease in the rate of GMSL rise thereafter (Fig. 1a). The local expression of relative sea-level rise across the UK and Ireland in the coming centuries shows large spatial variations from the global average that results from three main factors: (i) the ongoing effects on local sea level from GIA^31^; and the spatial patterns of change associated with the (ii) Greenland and (iii) Antarctic ice sheets^10,32^. Spatial patterns of sea-level rise at 2300 for Storylines A and B (Fig. 1b, c) are dominated by GIA with the characteristic pattern of vertical land uplift in the north and land subsidence in the south. For example, this effect substantially reduces the 2300 sea-level rise values for Belfast and Edinburgh to about 0.1–0.2 m, compared to 0.6–0.7 m for Cardiff and London in Storyline A. Storyline C also exhibits a strong imprint of GIA in the spatial pattern of sea-level rise (Fig. 1d) with values across the UK ranging from 3.5–4.5 m. The influence of spatial patterns associated with the Greenland and Antarctic ice sheets is more evident for Storylines H1 and H2 (Fig. 1e, f), with values at 2300 ranging from about 8.5–9.5 m and 16.5–17.5 m, respectively.

**Table 1:** Summary of sea-level rise storylines used in this study

Storyline	Scenario	Narrative Elements
A	RCP2.6/ SSP1-2.6	Strong emissions reductions over the 21st century and a low warming scenario. Low climate sensitivity and low sensitivity of the cryosphere and ocean to temperature rise. Plausible “best case scenario” for informing minimum adaptation requirements.
B	RCP4.5/ SSP2-4.5	Moderate warming scenario with moderate emissions reductions (approximately in line with current pledges under UNFCCC). Climate sensitivity close to IPCC AR6 best estimate^3^. Moderate response of the cryosphere and ocean to future warming.
C	RCP8.5/ SSP5-8.5	High warming scenario—policy “back-tracking” and/or carbon cycle feedbacks result in high greenhouse gas emissions throughout the 21st century and beyond. High climate sensitivity and response of the cryosphere and ocean to future warming.
H1	RCP8.5/ SSP5-8.5	High warming scenario—as Storyline C—with substantial ice sheet instability processes active for both Greenland and Antarctica (including Marine Ice Cliff Instability^8,47^)
H2	RCP8.5/ SSP5-8.5	High warming scenario—as Storyline C—with runaway ice sheet instability processes active in Antarctica (including Marine Ice Cliff Instability^8^), which overwhelmingly dominates future ice sheet contributions.

The flood mapping and impacts analysis presented here focuses on Storyline B as a baseline scenario (in the absence of further global greenhouse gas emissions pledges) and Storyline H2 as a “reasonable worst case” scenario for informing future coastal adaptation planning. We choose Storyline B as our baseline rather than Storyline A because the latter could be considered an overly optimistic basis for adaptation planning, given that it goes beyond current emissions pledges and assumes a weak sea-level response to future warming (Table 1). While we cannot give a robust likelihood for Storyline H2, we assess that it is broadly consistent with the UK National Risk Register definition of “reasonable worst case” of being “a challenging manifestation of the scenario after highly implausible scenarios are excluded” and note that IPCC AR6 states that this outcome cannot be ruled out under high emissions^3^. For a discussion of what constitutes “reasonable” in this context, please see Supplementary Note S1. GMSL rise at 2100 reaches approximately 0.6 m for Storyline B and 1.5 m for Storyline H2 (Fig. 1a), with large geographical variations across the UK and Ireland (Supplementary Fig. 1).

### Impacts of sea-level rise on UK coastal flood hazard

**Fig. 2:**
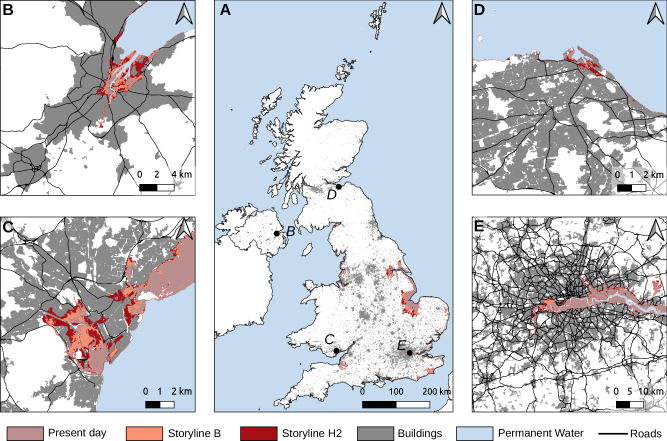
Flood maps including sea-level rise to 2100. **A** Flood inundation map for the UK based on the 1-in-200-year return period for the present day and with sea-level rise for Storylines B and H2 at 2100 in the absence of flood defences, as indicated in the figure legend. Panels **B**–**E** show local flood maps for Belfast, Cardiff, Edinburgh and London.

At a national scale, the changes in undefended inundation from a 1-in-200 year storm surge event (i.e., the basis of flood defence standards in the UK) shows modest changes with future sea-level rise at 2100 (Fig. 2A). The greatest areas of increased inundation are found for The Wash and the Humber Estuary on the east coast of England with a 13% national increase in total flood extent for Storyline B and 41% increase for Storyline H2 (Fig. 2A, Supplementary Table 3). To illustrate the local impacts of sea-level rise, we also present detailed mapping for the UK capital cities of Belfast (2B), Cardiff (2C), Edinburgh (2D) and London (2E), along with the present-day area at risk. For Storyline B by 2100, the additional areas of inundation are relatively modest. Given climate change and uncertainty tolerances within flood defence design^33^, it is likely that the real-world impacts of a ~ 0.5 m sea-level rise in areas protected by coastal flood defences would be limited. Nevertheless, many UK port facilities in southern England, which are necessarily outside of flood defences, will be impacted by a ~ 0.5 m rise in sea level due to the increase in extreme water levels during coastal surge events, and this may require additional protection measures to be adopted following port planning guidance and sector-specific climate change risk assessments^34,35^. For scenario H2 by 2100, the UK sea-level rise range of 1.2–1.5 m is more problematic, with large areas of west London, central Cardiff and infrastructure such as Belfast Airport within the zone inundated by the 1-in-200 year event. For H2, sea-level rise by 2100 is at the upper limit of what any present-day defences could plausibly protect against, so we can more definitively say these are areas that are not currently at risk but would be in the future.

**Fig. 3:**
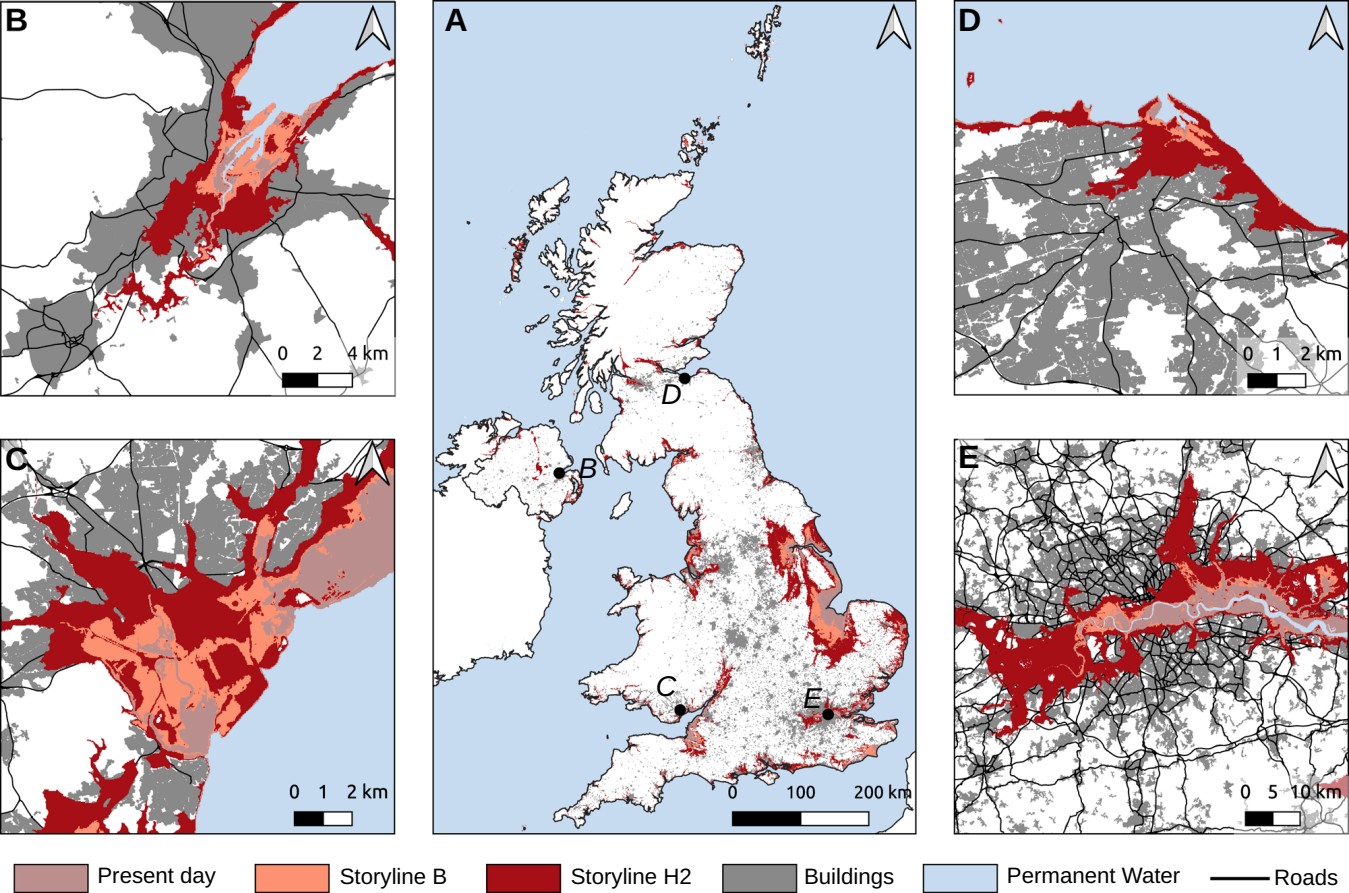
Flood maps including sea-level rise to 2300. **A** Flood inundation map for the UK based on the 1-in-200-year return period for the present day and with sea-level rise for Storylines B and H2 at 2300 in the absence of flood defences, as indicated in the figure legend. Panels **B**–**E** show local flood maps for Belfast, Cardiff, Edinburgh and London.

By 2300, both Storyline B and Storyline H2 imply dramatic changes for the UK coastal flood hazard (Fig. 3A). The Storyline B local relative sea-level rise of about 1.2–1.8 m across the UK translates, in the absence of flood defences, to a 56% increase in the flood area of the 1-in-200-year storm surge event (Fig. 1c, Supplementary Table 3). For Storyline H2, the local sea-level rise at 2300 ranges from about 16.5–17.5 m with more than a 300% increase in flood area for the 1-in-200-year surge event (Fig. 1f, Supplementary Table 3). For this amount of sea-level rise, present-day flood defences are largely irrelevant. At a national scale, for H2, it is The Wash and the Humber Estuary that show the largest change in area of land exposed to coastal flood hazard. However, we can now also see evidence of substantive changes in other parts of the United Kingdom, including the Thames estuary, Eastern Norfolk, North Somerset, Gloucestershire, Liverpool and the northwest coast of England. As before, Fig. 3 shows the details of inundation changes for the UK capital cities. Under Storyline B by 2300, some major public institutions, such as the Welsh parliament building (the Senedd) in Cardiff, now lie within the 200-year flood zone, along with major UK infrastructure such as Glasgow airport, Teesside chemical works and the port of Dover. In Storyline H2, many UK critical infrastructure locations are affected by flooding, including the M25 motorway (especially around Heathrow airport) and the financial district of the City of London. Nationally significant heritage, such as St. Paul’s Cathedral in London, lies within the flood zone, and large areas of west and north London are impacted by water depths in excess of 1 m. Along the east coast, the upland area of the Lincolnshire Wolds becomes an island during extreme floods, and coastal flooding here can even propagate inland as far as major cities such as Cambridge and York, both of which see their entire central areas flooded to depths of 1 m or more. Although we have not attempted to model changes in coastlines, Storyline H2 at 2300 would see large areas of low-lying land below the nominal high tide line and therefore permanently inundated.

**Fig. 4:**
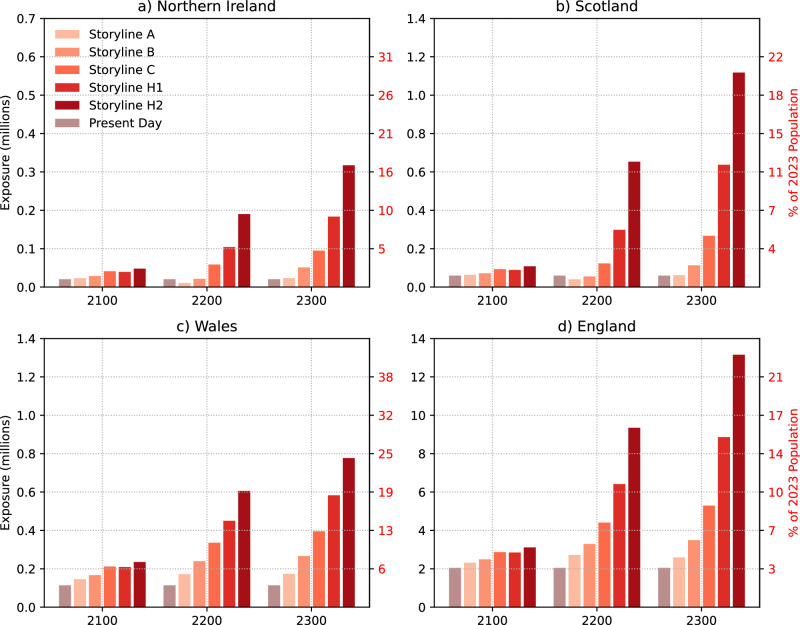
UK population exposure based on sea-level rise storylines. Population exposure for **a** England **b** Scotland **c** Wales **d** Northern Ireland at 2100, 2200 and 2300 for all sea-level rise storylines presented in the text, based on a 1-in-200-year return period. A 0.3 m threshold has been used to characterise the flood area (Supplementary Table 1). Numbers for alternative thresholds are available in the Supplementary Information.

The inundation maps have been combined with present-day UK population data^36^ to estimate the potential change in the population exposed to the 1-in-200 year flood event for each of the five storylines at 2100, 2200 and 2300 (Fig. 4). At 2100, all five storylines yield a modest increase in population exposure relative to the present day, with results indicating a low sensitivity to emissions pathways on this time-horizon. This outcome relates to the committed sea-level rise inherent to all storylines that is associated with past greenhouse gas emissions and the slow response of the ocean and cryosphere to surface warming^2^ (Supplementary Fig. 1). By 2200 there are much greater differences across the storylines associated with the broader range of sea-level rise outcomes (Supplementary Fig. 2) and even reductions in population exposure in Northern Ireland and Scotland for Storylines A and B where ongoing vertical land uplift associated with GIA overcomes the climate-change-induced sea-level rise. At 2300, the differences across storylines are even more pronounced, with the percentage of population exposed to the 1-in-200-year coastal flood extent exceeding 20% for England, Scotland and Wales under the H2 storyline (compared to between 1–3% in the present day).

Although defences have not been explicitly modelled, we can infer from the inundation modelling when these defences would be overtopped for the various scenarios modelled. This can help identify tipping points in the scenario-time horizon space where significant investment or adaptation may be required to limit exposure to coastal flood events. The inundation maps have been intersected with defence crest elevations from the Environment Agency Asset Information Management System (AIMS) dataset^37^ to assess the proportion of defences in England for which the modelled water surface elevation exceeds their height. Under Storyline B, in 2100, <1% of defences are modelled to have a maximum water level exceeding the defence height by 1 m or more. This increases to 27% by 2200 and 35% by 2300. Comparatively, under Storyline H2, in 2100, 10% of defences are modelled to have a maximum water level exceeding the defence height by 1 m or more, increasing to nearly 100% by 2200. Further information on this approach is provided in Supplementary Note S3 with a table of results presented in Supplementary Table 8.

## Discussion

Current UK national planning guidance for sea-level rise focuses on the coming century only and includes allowances that range from about 1.0 to 1.6 m over the period 2000 to 2125, depending on geographic location (https://www.gov.uk/guidance/flood-risk-assessments-climate-change-allowances). Where it is appropriate to use a “credible maximum scenario”, planners are advised to use the H + + sea-level rise allowance value of 1.9 m^38^, which represents a value for the end of the 21st century—no guidance is provided beyond this time-horizon. In addition, residual uncertainty allowances (typically a few 10 s of cm) are implemented when building flood defences in the UK, affording some extra margin of protection^33^. At 2100, this combination of climate scenarios and residual uncertainty allowances should therefore likely accommodate the values of sea-level rise for the UK across all storylines that have peak values of about 1.5 m (Supplementary Fig. 1). Under storyline B, the 2100 population exposed to flooding increases by 25%, which is slightly below the 27% increase in properties at risk by 2069 predicted by the most recent UK National Flood Risk Assessment^39^ (NaFRA2). Therefore, our exposure storylines are broadly consistent while being conservative with respect to UK government projections this century. The estimated changes in UK population exposure are similar to previous studies under similar climate scenarios and flood defence assumptions, with an increase of approximately 0.5 million for Storyline B and approximately 1 million for Storyline C by 2100^40^. Under Storyline B it is unlikely that hard flood defence adaptation limits will be reached by 2100 (Supplementary Table 8). This is also the case for the majority of defences for Storyline H2, although significant levels of investment would be required to increase the height of defences over the coming century (Supplementary Table 8).

Beyond 2100, the sea-level storylines diverge markedly with large differences in the associated flood impacts by 2300 (Figs. 1, 3). Storyline A clearly illustrates the huge potential for avoided sea-level rise through swift and substantive global greenhouse gas emissions reductions consistent with Paris Agreement targets, with exposure increasing by only 28%. Conversely, Storyline H2 illustrates the vast threat of sea-level rise for the UK if ice sheet instability processes are triggered with values that far exceed the current UK H++ credible maximum scenario of 1.9 m within a few decades of 2100 and a five-fold increase in population exposure by 2300 - vastly outside the range reported in NaFRA2^39^. While the UKCP18 national projections included sea-level rise information to 2300, this did not include an update to H++ or any equivalent credible maximum scenario on this time-horizon.

Our results show many instances of major UK infrastructure moving into flood risk areas as the sea level continues to rise beyond 2100. Increased exposure to coastal flooding is identified across all storylines. Under Storyline B, approximately half of England’s coastal flood defences would need investment over the subsequent 200 years to maintain defence standards, with a small number potentially being beyond hard adaptation limits (Supplementary Table 8). Under Storyline H2, >99% of flood defences could already be beyond hard adaptation limits by 2200 (Supplementary Table 8). The increased population exposure to the 1-in-200-year flood event under the H2 storyline from 3 million to almost 10 million between 2100 and 2200 implies widespread disruption to the economy and society, with challenges compounded by potentially reaching hard adaptation limits (Supplementary Table 8). This scenario would require high levels of investment for both new and existing flood defences, potential movement of communities away from coastlines, and put critical infrastructure and sites of national strategic importance at high risk of disruption.

It is therefore inevitable that a combination of additional or enhanced flood defences and managed retreat will be necessary to mitigate against post-2100 flood risk. Stakeholders who only consider changes over the coming century may have a false impression of the true risk landscape, given the potential for post-2100 acceleration of sea-level rise. Planning of long-lived infrastructure and future settlements must therefore be cognisant of the full range of possible outcomes for multi-century and longer-term committed sea-level rise to avoid costly future interventions.

There is a clear need for continuous monitoring of sea-level rise with a particular emphasis on the ice sheets to understand the observed trajectory compared to expectations across a broad range of possible outcomes. This will allow as much time as possible to prepare for and react to extreme future sea-level rise should one of the more extreme scenarios be realised.

The Thames Estuary 2100 project^41^ takes an adaptation pathways approach to managing future flood risk for London and planning of future adaptation measures throughout the estuary^42,43^. This approach triggers different adaptation options as various sea-level rise “milestones” are met. Combining our storylines with adaptive pathways approaches could form a powerful tool for reflexive learning and promoting robust adaptation decision-making^42^. However, previous work has suggested that the engineering limits to adaptation for the Thames Estuary would be surpassed at approximately 5 m of sea-level rise^44^, which raises major questions for the city of London under the H1 and H2 storylines presented here. For storylines H1 or H2, London reaches 5 m of sea-level rise at around 2150 or 2200 (Supplementary Table 4) with corresponding widespread flood implications across the UK. For multiple metres of sea-level rise, maintaining current coastlines would prove extremely challenging for most of the UK and would therefore likely necessitate large-scale movement of settlements, population and infrastructure away from the coast. Given the high associated costs and long planning time-horizons, it is vital to consider potential peak rates of future UK sea-level rise in adaptation planning processes in order to anticipate decision lead-times. Due to the country-wide connectivity of vital infrastructure, it is imperative that full cost-benefit analyses^45,46^ are undertaken at the national scale to fully understand the economic, political and societal impacts of coastal flooding under such extreme future sea-level rise scenarios. This type of assessment would require a set of plausible scenarios that include feedback between population dynamics and the evolving flood hazard under adaptation measures and future sea-level rise that extend into the coming centuries.

Overall, our results clearly illustrate the need to consider multi-century time-horizons to fully appreciate the scale of the coastal adaptation challenge, given deep uncertainty in projections of future sea-level rise. The “reasonable worst case scenario” (storyline H2) implies a substantively altered UK coastline in the coming centuries, with a need for large-scale movement of population, settlements and infrastructure away from the coast. However, the results also indicate how the worst impacts of sea-level rise could be avoided in the coming centuries if effective global action can be taken to minimise future global greenhouse gas emissions. The key policy implications of our study are to inform near-term adaptation planning for the period out to 2100 and further motivate rapid and deep reductions in global greenhouse gas emissions to mitigate against the risk of accelerated sea-level rise from ice sheet instability processes in the centuries that follow.

## Methods

The sea-level rise storylines used in this study are based on those presented in Palmer et al.^10^ and the reader is referred there for further details. The five storylines each represent a singular and continuous trajectory of future sea-level rise drawn from the underlying sets of large Monte Carlo simulations developed for UKCP18 under the RCP scenarios^11,12^. Each Monte Carlo set includes 450,000 simulations of the following contributions to GMSL rise: (i) global thermosteric sea-level rise; (ii) mass addition from Antarctica; (iii) mass addition from Greenland; (iv) mass addition from mountain glaciers; (v) changes in land water storage. The storylines are generated following a two-step process that is designed to provide close consistency with several literature-based estimates of GMSL rise and the underlying process-based contributions. First, the Monte Carlo set is filtered to retain only those members that match the target GMSL rise at the given time-horizon. Second, a single Monte Carlo member is selected that provides the best fit to the individual contributions (i)–(v). Storyline A is based on the RCP2.6 Monte Carlo simulations selected for consistency with the lower bound of the *likely* range (i.e. the 17th percentile) of the IPCC AR6 GMSL projections under SSP1-2.6 at 2150. Storyline B is based on the RCP4.5 Monte Carlo simulations and selected to be consistent with the central estimate (i.e. the 50th percentile) of the IPCC AR6 GMSL projections under SSP2-4.5 at 2150. Storyline C is based on the RCP8.5 Monte Carlo simulations and selected for consistency with the upper bound of the *likely* range (i.e. the 83rd percentile) of IPCC AR6 GMSL projections under SSP5-8.5 at 2150. Storylines H1 and H2 are both based on the RCP8.5 Monte Carlo simulations with the Antarctica component replaced by the DeConto et al.^8^ simulations that represent the marine ice cliff instability (MICI) mechanism for Antarctica^47^. H1 is selected for consistency with the high-end estimate of GMSL at 2300 developed by van de Wal et al.^48^. H2 is consistent with the IPCC AR6 SSP5-8.5 *low confidence* projections, 83rd percentile for GMSL rise at 2300^2^. The GMSL storylines are localised for the UK by first combining timeseries of (ii)–(v) with the corresponding spatial patterns of Gravity, Rotation and solid-earth Deformation (GRD) to provide spatially complete information for across the domain of the UK and Ireland at 2100, 2200 and 2300. Secondly, timeseries of (i) are converted to a representative UK-average of the sterodynamic sea-level change using a spatially uniform regression coefficient based on the scenario-specific central estimates from UKCP18. These values are: 1.372 for SSP1-2.6/RCP2.6 (used for Storyline A); 1.390 for SSP2-4.5/RCP4.5 (used for Storyline B), and 1.465 for SSP5-8.5/RCP8.5 (used for Storyline C, H1, H2). All regression coefficients result in a time-invariant amplification of the global signal that is largest for the SSP5-8.5/RCP8.5 scenario. Further justification of this approach is presented in Supplementary Note S2. Finally, the spatial contribution to local sea-level change from Glacial Isostatic Adjustment (GIA) is added by assuming a linear rate of change and using the central estimate from the UKCP18 sea-level projections^11,31^.

Projections of future flood hazard are based on the UK flood inundation model of Bates et al.^23^. This simulates floodplain flow by solving the local inertial form of the shallow water equations in two dimensions (2D) for a uniform grid using the numerical approach outlined in previous studies^49,50^. Channel flow is treated as a subgrid scale process solved in one dimension (1D) using the method of Neal et al.^34^ This allows river channels of any size to be represented, irrespective of the resolution of the overlying floodplain grid. The 2D model is run over the whole UK at 1 arcsecond resolution (20–25 m), with terrain elevations taken mostly from 10 cm vertical accuracy airborne laser altimetry (LiDAR) data, for which the coverage in the UK is approximately 70% and concentrated in lowland zones. The vertical datum is Ordnance Datum Newlyn in Great Britain and Ordnance Datum Belfast in Northern Ireland. Channel locations for the 1D component of the model are taken from UK Ordnance Survey data, with channel widths estimated based on hydraulic geometry relationships and channel bathymetry approximated using the gradually varied flow solver methodology of Neal et al.^51^ and channel friction of 0.04. We assume a bankfull return period of 1 in 2 years for natural alluvial channels. A fixed value of 0.06 is applied for the floodplain friction parameter in the model. Coastal boundary conditions for the model combine the storyline-based sea-level rise projections with the Environment Agency coastal flood boundary conditions dataset^52^ which defines the probability-magnitude of meteorologically-driven coastal extreme water levels above mean sea level around the UK. These extreme water heights were combined with the relevant storm surge profile for the stretch of coastline (as defined by the Environment Agency Design Surge Profile dataset), a time-varying profile based on historic tide gauge data and an estimate of wave setup (calculated using ERA5 reanalysis data^53^) to produce an extreme water height time series for the storm surge event that was simulated by the inundation model. Further details of the boundary condition and model discretisation can be found in Bates et al.^23^ who showed that this model could skilfully predict present-day flood areas and water depths when compared to UK Government official flood maps and post-event water depth observations.

For this study, we re-simulated the inundation hydrodynamics of a 1-in-200-year extreme event (based on the central estimate) for UK coastal regions with peak water elevations increased to represent the impact of the five sea-level rise storylines 2100, 2200 and 2300. We simulated events with a return period of 1-in-200 years, as this is the current standard of protection for coastal flood defences in the UK and is typically presented in UK national flood risk maps. We use a version of our model without flood defences to provide a consistent baseline since we do not know the future trajectory of protection standards and because many of the storylines would exceed current design standards along most of the coastline outside London. Flood defences will mitigate the extent of flooding even when overwhelmed, however we chose to omit this second-order factor rather than include uncertain and spatially incomplete defence locations and heights from existing databases in the model. Readers should refer to Bates et al.^20^ and Environment Agency data for near-term hazard estimates that include existing flood defence standards. For each individual model, the maximum water level was defined and then converted into a water level time series through the event using standard tidal cycles. Our approach is supported by previous studies that have shown that mean sea level, tides and surge combine linearly, to a good approximation^11,54^. We acknowledge that the modelling approach neglects coastline and bathymetry changes associated with erosion and sedimentation processes, which could further modify extreme coastal water levels through associated tide and surge changes. These aspects are likely to be increasingly important under large degrees of future sea-level rise and warrant further research.

### Reporting summary

Further information on research design is available in the Nature Portfolio Reporting Summary linked to this article.

## Supplementary information

Supplementary Information

Reporting Summary

Transparent Peer Review file

## Data Availability

The data generated in this study have been deposited in the Zenodo database under Record ID 18862415, available at 10.5281/zenodo.18862415. This archive also includes the essential third-party datasets: (i) Environment Agency extreme water level dataset; (ii) the Digital Elevation Model; and (iii) the WorldPop population dataset. The sea-level rise storyline data used in this study are available in the Zenodo database under Record ID 19008851, available at 10.5281/zenodo.17312508. The coastal boundary condition dataset can be downloaded from the Environment Agency website directly at https://environment.data.gov.uk/dataset/84a5c7c0-d465-11e4-b0bd-f0def148f590. Supporting documentation on how to use this data can be found on the UK government website: https://www.gov.uk/flood-and-coastal-erosion-risk-management-research-reports/coastal-flood-boundary-conditions-for-the-mainland-uk-coasts-and-islands. LiDAR data can be found on the UK Government websites at the following locations: England https://environment.data.gov.uk/dataset/09ea3b37-df3a-4e8b-ac69-fb0842227b04, Scotland https://remotesensingdata.gov.scot/data#/list, Wales https://datamap.gov.wales/maps/lidar-data-download/. Northern Ireland https://admin.opendatani.gov.uk/dataset/?tags=Elevation&tags=Digital+Terrain+Model&_tags_limit=0. OS Open Terrain can be downloaded at https://osdatahub.os.uk/data/downloads/open/Terrain50. Tide gauge data for the UK can be found on the BoDC website https://www.bodc.ac.uk/data/. ERA-5 reanalysis data can be downloaded on the Copernicus website here: https://cds.climate.copernicus.eu/datasets/reanalysis-era5-single-levels. WorldPOP data is available at https://www.worldpop.org/. Merit-Hydro is available for academic purposes at https://hydro.iis.u-tokyo.ac.jp/~yamadai/MERIT_Hydro/.

## Code availability

The code used to generate Figs. 1, 4 (and associated Supplementary Figs.) are publicly available at 10.5281/zenodo.18862415. Figures 2, 3 were produced in QGIS using version 3.22.4. The QGIS software is freely available from https://qgis.org/. The LISFLOOD-FP code used for the flood inundation modelling is open source and available at https://zenodo.org/records/13121102.
