# Supplementary material for: Quantifying UK coastal flood exposure under future sea-level rise to 2300
Source: Nat Commun. 2026 Jul 21;17:5882. doi: 10.1038/s41467-026-74982-1 (PMC13389417; doi:10.1038/s41467-026-74982-1)
Supplement: Supplementary file 1 — Supplementary Information [file 41467_2026_74982_MOESM1_ESM.pdf]

SUPPLEMENTARY INFORMATION

Quantifying UK coastal flood exposure under future sea-level rise to 2300

M.D Palmer*[1,2], J. Savage[3], P.D. Bates[3,4] and J. Neal[3,4]

1: Met Office Hadley Centre, Exeter UK

2: School of Earth Sciences, University of Bristol, UK

3: Fathom, Bristol, UK

4: School of Geographical Sciences, University of Bristol, UK

*corresponding author: matthew.palmer@metoffice.gov.uk

Supplementary Fig. 1: sea-level rise storylines and 2100 spatial patterns. a) Time series of global mean sea-level (GMSL) rise for the five storylines presented in Palmer et al[1] as indicated in the figure legend. The grey shaded region shows the overall IPCC AR6 *likely* range (i.e. the central two-thirds probability distribution) across the SSP1-2.6 and SSP5-8.5 greenhouse gas emissions scenarios to 2150. The grey dotted line shows the IPCC AR6 low-likelihood high-impact storyline presented in the Working Group I Summary for Policymakers[2]. b)-f) the spatial pattern of relative sea-level rise at 2100 associated with each storyline. All sea-level rise projections are expressed relative to the 1986-2005 average.

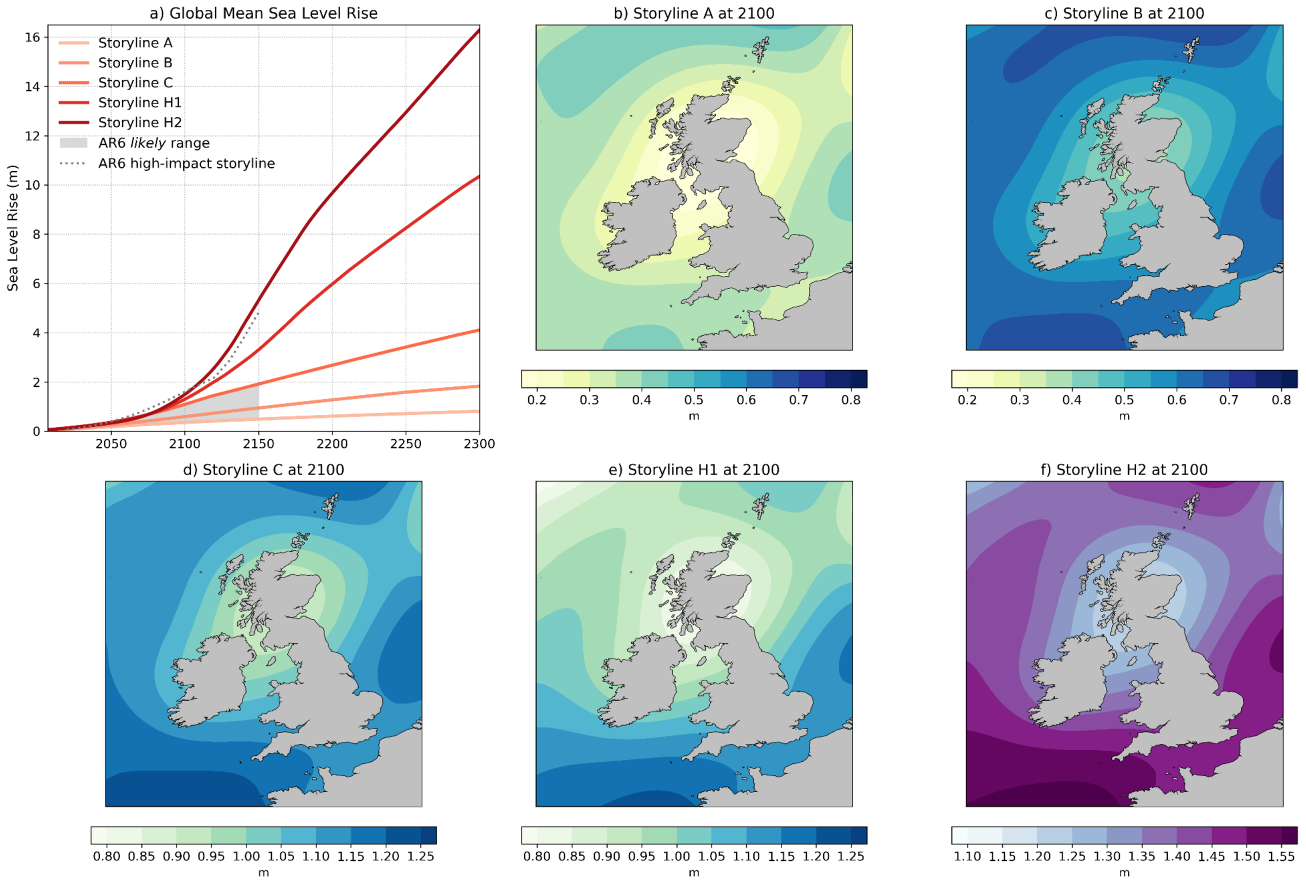

Supplementary Fig. 2: sea-level rise storylines and 2200 spatial patterns. a) Time series of global mean sea-level (GMSL) rise for the five storylines presented in Palmer et al[1] as indicated in the figure legend. The grey shaded region shows the overall IPCC AR6 *likely* range (i.e. the central two-thirds probability distribution) across the SSP1-2.6 and SSP5-8.5 greenhouse gas emissions scenarios to 2150. The grey dotted line shows the IPCC AR6 low-likelihood high-impact storyline presented in the Working Group I Summary for Policymakers[2]. b)-f) the spatial pattern of relative sea-level rise at 2200 associated with each storyline. All sea-level rise projections are expressed relative to the 1986-2005 average.

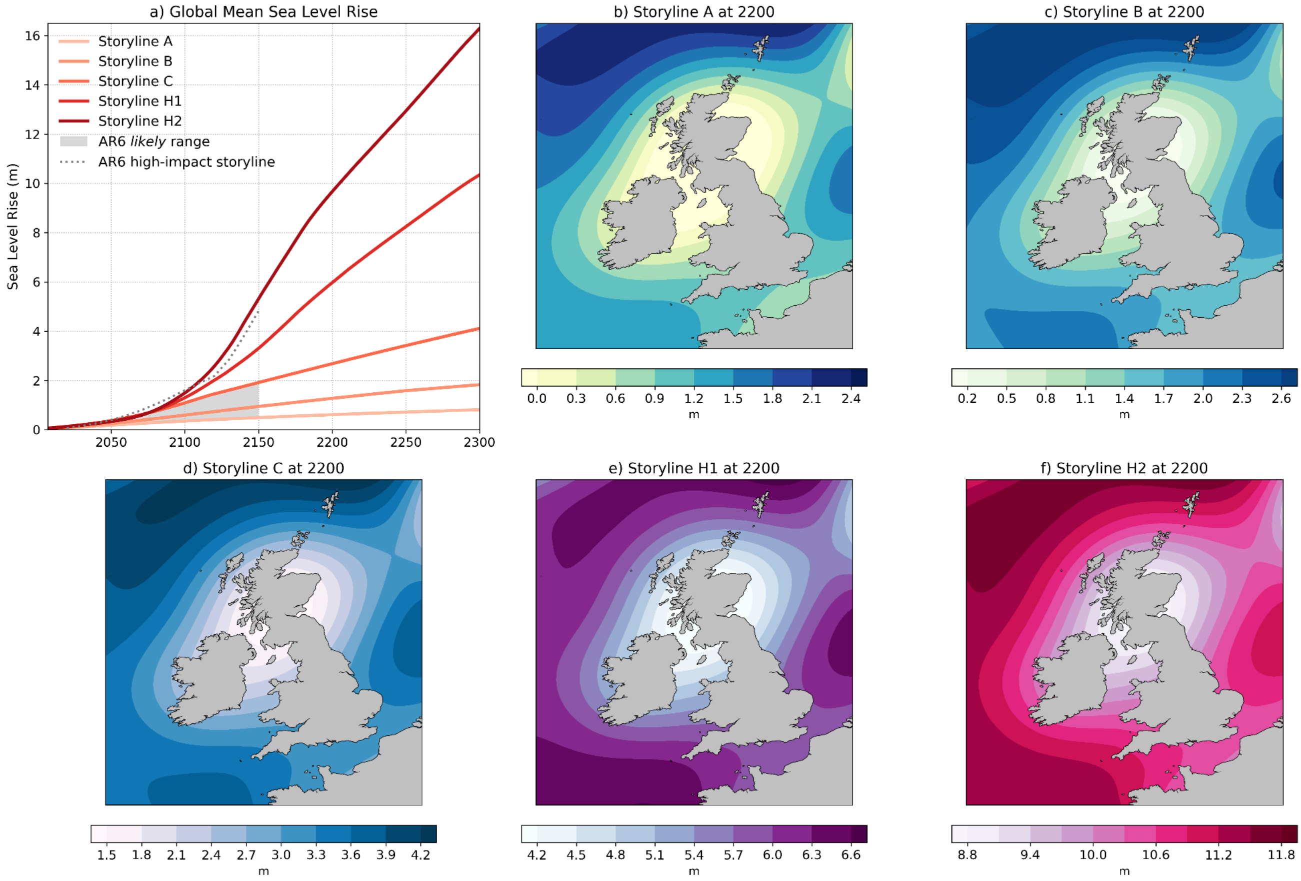

Supplementary Fig. 3: Flood maps including sea-level rise to 2200. A) Flood inundation maps for the UK based on the 1-in-200-year return period for the present day and with sea-level rise for Storylines B and H2 at 2200 in the absence of flood defences, as indicated in the figure legend. Panels B)-E) show local flood maps for Belfast, Cardiff, Edinburgh and London.

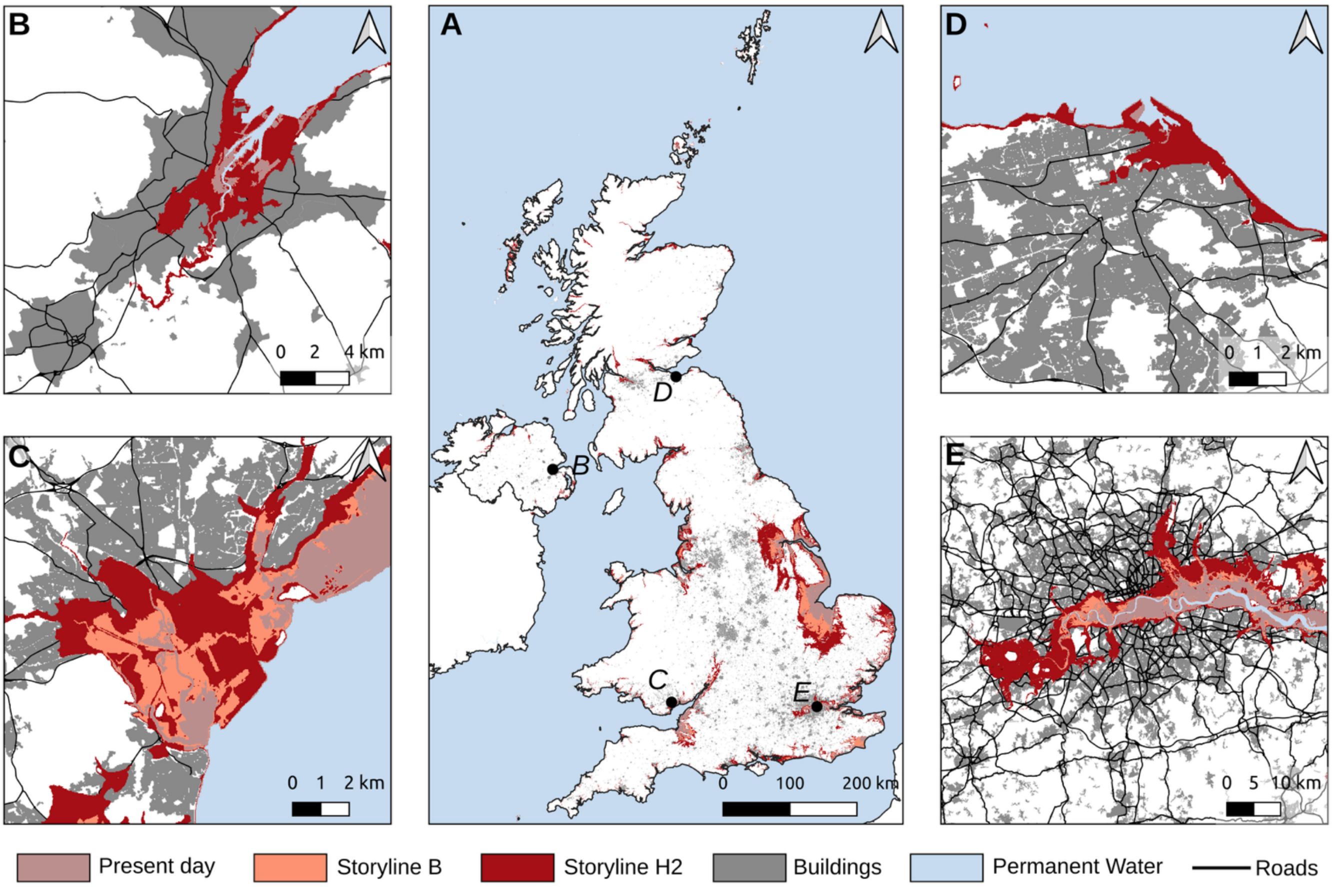

Supplementary Table 1: Population exposure based on 0.3 m threshold. The UK population exposed to the 1-in-200-year return period event in the absence of coastal flood defences using a 0.3 m threshold. See supplementary information for alternative thresholds. All results are reported to 2 significant figures.

Storyline	Time Horizon	Exposure (millions) for 0.3 m threshold				
		England	Wales	Scotland	Northern Ireland	TOTAL
Story A	2100	2.3	0.15	0.064	0.023	2.6
Story A	2200	2.7	0.17	0.040	0.011	2.9
Story A	2300	2.6	0.17	0.062	0.023	2.9
Story B	2100	2.5	0.17	0.071	0.029	2.8
Story B	2200	3.3	0.24	0.055	0.022	3.6
Story B	2300	3.5	0.27	0.11	0.051	3.9
Story C	2100	2.9	0.21	0.094	0.041	3.2
Story C	2200	4.4	0.34	0.12	0.059	4.9
Story C	2300	5.3	0.40	0.27	0.095	6.0
Story H1	2100	2.8	0.21	0.089	0.039	3.2
Story H1	2200	6.4	0.45	0.30	0.10	7.3
Story H1	2300	8.9	0.58	0.64	0.18	10
Story H2	2100	3.1	0.24	0.11	0.048	3.5
Story H2	2200	9.3	0.61	0.65	0.19	11
Story H2	2300	13	0.78	1.1	0.32	15
Present Day		2.0	0.11	0.060	0.020	2.2

Supplementary Table 2: Flood areas based on sea level-rise storylines. The flood area associated with the 1-in-200-year return period event at 2100, 2200 and 2300 for the five storylines described in the main text. All results are reported to 2 significant figures.

Storyline	Time Horizon	Flood Area (km^2)			
		0.0m Threshold	0.1m Threshold	0.3m Threshold	1.0m Threshold
Story A	2100	7100	6900	6600	5200
Story A	2200	8100	8000	7700	6500
Story A	2300	7900	7800	7500	6200
Story B	2100	7600	7500	7200	5900
Story B	2200	9600	9500	9300	8200
Story B	2300	10000	10000	9900	8900
Story C	2100	8800	8700	8400	7200
Story C	2200	12000	12000	12000	11000
Story C	2300	15000	15000	14000	13000
Story H1	2100	8700	8600	8300	710087
Story H1	2200	17000	17000	16000	15000
Story H1	2300	21000	21000	21000	20000
Story H2	2100	9400	9300	9000	7800
Story H2	2200	22000	22000	22000	21000
Story H2	2300	29000	29000	29000	28000
Present Day		6600	6600	6400	5000

Supplementary Table 3: Percentage change in flood area based on sea-level rise storylines. The percentage change in flood area (compared to present day) associated with the 1-in-200-year return period event at 2100, 2200 and 2300 for the five storylines described in the main text. All results are reported to 2 significant figures.

Storyline	Time Horizon	Flood Area (% Increase)			
		0.0m Threshold	0.1m Threshold	0.3m Threshold	1.0m Threshold
Story A	2100	5.4	3.2	3.5	5.6
Story A	2200	21	19	21	32
Story A	2300	18	16	17	25
Story B	2100	14	12	13	19
Story B	2200	44	42	46	66
Story B	2300	54	53	56	79
Story C	2100	31	29	31	45
Story C	2200	86	84	90	130
Story C	2300	120	120	130	170
Story H1	2100	30	28	30	43
Story H1	2200	150	150	160	210
Story H1	2300	220	220	230	310
Story H2	2100	40	39	41	58
Story H2	2200	230	230	240	320
Story H2	2300	330	330	350	460
Present Day		0.0	0.0	0.0	0.0

Supplementary Table 4: Timing of sea-level rise milestones. The timing of sea-level rise milestones at UK capital cities for the five storylines described in the main text.

Storyline / Location	Year that sea-level rise milestone is first reached							
	0.5 m	1.0 m	1.5 m	2.0 m	2.5 m	3.0 m	4.0 m	5.0 m
Story A – Belfast	-	-	-	-	-	-	-	-
Story A – Cardiff	2176	-	-	-	-	-	-	-
Story A – Edinburgh	-	-	-	-	-	-	-	-
Story A – London	2160	-	-	-	-	-	-	-
Story B – Belfast	2115	2229	-	-	-	-	-	-
Story B – Cardiff	2087	2163	2249	-	-	-	-	-
Story B – Edinburgh	2124	2250	-	-	-	-	-	-
Story B – London	2085	2159	2242	-	-	-	-	-
Story C – Belfast	2068	2104	2140	2175	2211	2248	-	-
Story C – Cardiff	2059	2094	2123	2155	2186	2218	2285	-
Story C – Edinburgh	2070	2107	2144	2180	2217	2256	-	-
Story C – London	2059	2093	2122	2153	2184	2215	2281	-
Story H1 – Belfast	2082	2108	2125	2139	2151	2161	2181	2203
Story H1 – Cardiff	2069	2098	2116	2130	2142	2153	2172	2191
Story H1 – Edinburgh	2084	2110	2127	2141	2153	2163	2183	2207
Story H1 – London	2067	2096	2114	2129	2141	2152	2171	2190
Story H2 – Belfast	2075	2094	2106	2116	2123	2129	2139	2149
Story H2 – Cardiff	2067	2088	2102	2112	2120	2126	2137	2146
Story H2 – Edinburgh	2076	2095	2108	2117	2124	2130	2140	2150
Story H2 – London	2066	2088	2102	2112	2120	2126	2137	2146

Supplementary Table 5: Population exposure based on 0.0 m threshold. The UK population exposed to the 1-in-200-year return period event in the absence of coastal flood defences using a 0.0 m threshold. All results are reported to 2 significant figures.

Storyline	Time Horizon	Exposure (millions) for 0.0 m threshold				
		England	Wales	Scotland	Northern Ireland	TOTAL
Story A	2100	2.5	0.17	0.074	0.029	2.8
Story A	2200	2.9	0.20	0.046	0.013	3.2
Story A	2300	2.8	0.20	0.072	0.030	3.1
Story B	2100	2.7	0.19	0.083	0.036	3.0
Story B	2200	3.5	0.26	0.064	0.027	3.8
Story B	2300	3.7	0.28	0.13	0.057	4.2
Story C	2100	3.1	0.23	0.11	0.048	3.5
Story C	2200	4.6	0.35	0.14	0.065	5.1
Story C	2300	5.5	0.41	0.29	0.099	6.3
Story H1	2100	3.0	0.23	0.11	0.047	3.4
Story H1	2200	6.6	0.46	0.32	0.11	7.5
Story H1	2300	9.0	0.59	0.65	0.19	10
Story H2	2100	3.3	0.25	0.12	0.054	3.7
Story H2	2200	9.5	0.62	0.67	0.20	11
Story H2	2300	13	0.79	1.14	0.32	16
Present Day		2.2	0.13	0.066	0.024	2.4

Supplementary Table 6: Population exposure based on 0.1 m threshold. The UK population exposed to the 1-in-200-year return period event in the absence of coastal flood defences using a 0.1 m threshold. All results are reported to 2 significant figures.

Storyline	Time Horizon	Exposure (millions) for 0.1 m threshold				
		England	Wales	Scotland	Northern Ireland	TOTAL
Story A	2100	2.4	0.16	0.071	0.027	2.7
Story A	2200	2.8	0.19	0.044	0.012	3.1
Story A	2300	2.7	0.19	0.069	0.028	3.0
Story B	2100	2.6	0.18	0.079	0.034	2.9
Story B	2200	3.4	0.25	0.062	0.026	3.8
Story B	2300	3.6	0.28	0.12	0.055	4.1
Story C	2100	3.0	0.23	0.11	0.046	3.4
Story C	2200	4.5	0.35	0.13	0.063	5.1
Story C	2300	5.4	0.40	0.28	0.097	6.2
Story H1	2100	3.0	0.22	0.10	0.044	3.3
Story H1	2200	6.5	0.46	0.31	0.11	7.4
Story H1	2300	9.0	0.59	0.65	0.19	10
Story H2	2100	3.2	0.25	0.12	0.052	3.7
Story H2	2200	9.5	0.61	0.67	0.19	11
Story H2	2300	13	0.78	1.13	0.32	15
Present Day		2.2	0.13	0.066	0.024	2.4

Supplementary Table 7: Population exposure based on 1.0 m threshold. The UK population exposed to the 1-in-200-year return period event in the absence of coastal flood defences using a 1.0 m threshold. All results are reported to 2 significant figures.

Storyline	Time Horizon	Exposure (millions) for 1.0 m threshold				
		England	Wales	Scotland	Northern Ireland	TOTAL
Story A	2100	1.8	0.092	0.042	0.012	2.0
Story A	2200	2.3	0.12	0.030	0.007	2.4
Story A	2300	2.1	0.12	0.041	0.012	2.3
Story B	2100	2.0	0.12	0.047	0.015	2.2
Story B	2200	2.8	0.18	0.039	0.011	3.0
Story B	2300	3.0	0.22	0.074	0.033	3.3
Story C	2100	2.4	0.16	0.061	0.023	2.7
Story C	2200	4.0	0.30	0.083	0.042	4.4
Story C	2300	4.8	0.36	0.21	0.085	5.5
Story H1	2100	2.4	0.16	0.058	0.022	2.6
Story H1	2200	6.0	0.43	0.24	0.094	6.8
Story H1	2300	8.4	0.56	0.59	0.17	9.8
Story H2	2100	2.6	0.18	0.070	0.029	2.9
Story H2	2200	8.9	0.59	0.61	0.18	10
Story H2	2300	13	0.76	1.1	0.31	15
Present Day		1.5	0.070	0.040	0.011	1.6

Supplementary Note S1: Definition of "reasonable worst case"

What constitutes a reasonable worst case in any given circumstance is essentially a value judgement. However, the UK National Risk Register[3] attempts to formalize this definition by stating that the reasonable worst case is "not a prediction of what is likely to happen" but represents a "challenging manifestation of the scenario after highly implausible scenarios are excluded". The National Risk Register tries to classify the annual percentage chance of different threats into one of five categories from >25% (unlikely to almost certain) to <0.2% (remote chance), with indicative economic losses for the latter being of the order of tens of billions GBP. These definitions are derived from the probability yardstick[4] developed in the 2000s for use by the UK intelligence community for communicating uncertainties in assessments. Evaluations are made using both quantitative data (where possible) and expert judgement (where not), with the latter approach more typical for lower probability risks. The likelihood of Marine Ice Cliff Instability (MICI)[5] occurring at scale by 2300, which would create scenarios of extreme sea-level rise, is currently poorly known but cannot be credibly ruled out. The UK's existing "reasonable worst case" scenarios already include similar low probability yet poorly-known events and it is thus clear that scenarios H1 and H2 fall within the scope of the National Risk Register definition. For this reason, we designate H2 as a reasonable worst case scenario for UK sea-level rise by 2300.

Supplementary Note S2: Approach to local sterodynamic sea-level rise

Following the methods presented in UKCP18[6], a scenario-specific space-and-time invariant regression coefficient is used to estimate the local sterodynamic sea change for each storyline. For each CMIP5 model the linear best fit between local sterodynamic sea-level change (CMIP variables: zos + zostoga) and global thermosteric sea-level change (CMIP variable: zostoga) is computed for every UK-adjacent model grid box (see Palmer et al[6] for further details). For each scenario, we take an average across all CMIP5 model regression coefficients that ensures each model is weighted equally. This results in a single regression coefficient for each scenario that represents the average for the UK across the CMIP5 ensemble. The assumption of spatial invariance was originally made in UKCP18 because the underlying CMIP5 global climate model (GCM) simulations have limited horizontal and vertical resolution and are missing key processes, such as tides[7]. Therefore, it was concluded that any spatial variations around the UK coast were unlikely to be robust and that an assumption of spatial uniformity was more appropriate[6]. Subsequently, Hermans et al[8] have shown that downscaling of CMIP5 GCM simulations results in essentially spatially uniform patterns of sterodynamic sea-level change round the UK coastline.

Under UKCP18, scenario-specific regression coefficients for each RCP scenario were computed on the basis of a linear fit between global thermosteric sea-level rise and local sterodynamic sea-level rise for CMIP5 model simulations over the period 2007-2100 (Figure S1). The same process was carried out for each CMIP5 GCM grid box adjacent to the UK coastline. Given the assumption of spatial invariance noted above, a central estimate value was used across all available CMIP5 UK coastal grid boxes. It was assumed that these regression coefficients were representative of the time-horizon out to 2300, which is a reasonable first-order approximation in most cases, as illustrated for the available CMIP5 simulations under the RCP4.5 scenario (Figure S2). Further details are available in Palmer et al[6].

Supplementary Fig. 4: Regression relationships between local sterodynamic sea level and global thermal expansion for Newlyn for the period 2007-2100. Relationships are plotted for all available simulations: RCP2.6 (blue); RCP4.5 (cyan); and RCP8.5 (red). For comparison, the dotted line shows the 1:1 relationship. Reproduced from the UKCP18 Marine Report[6].

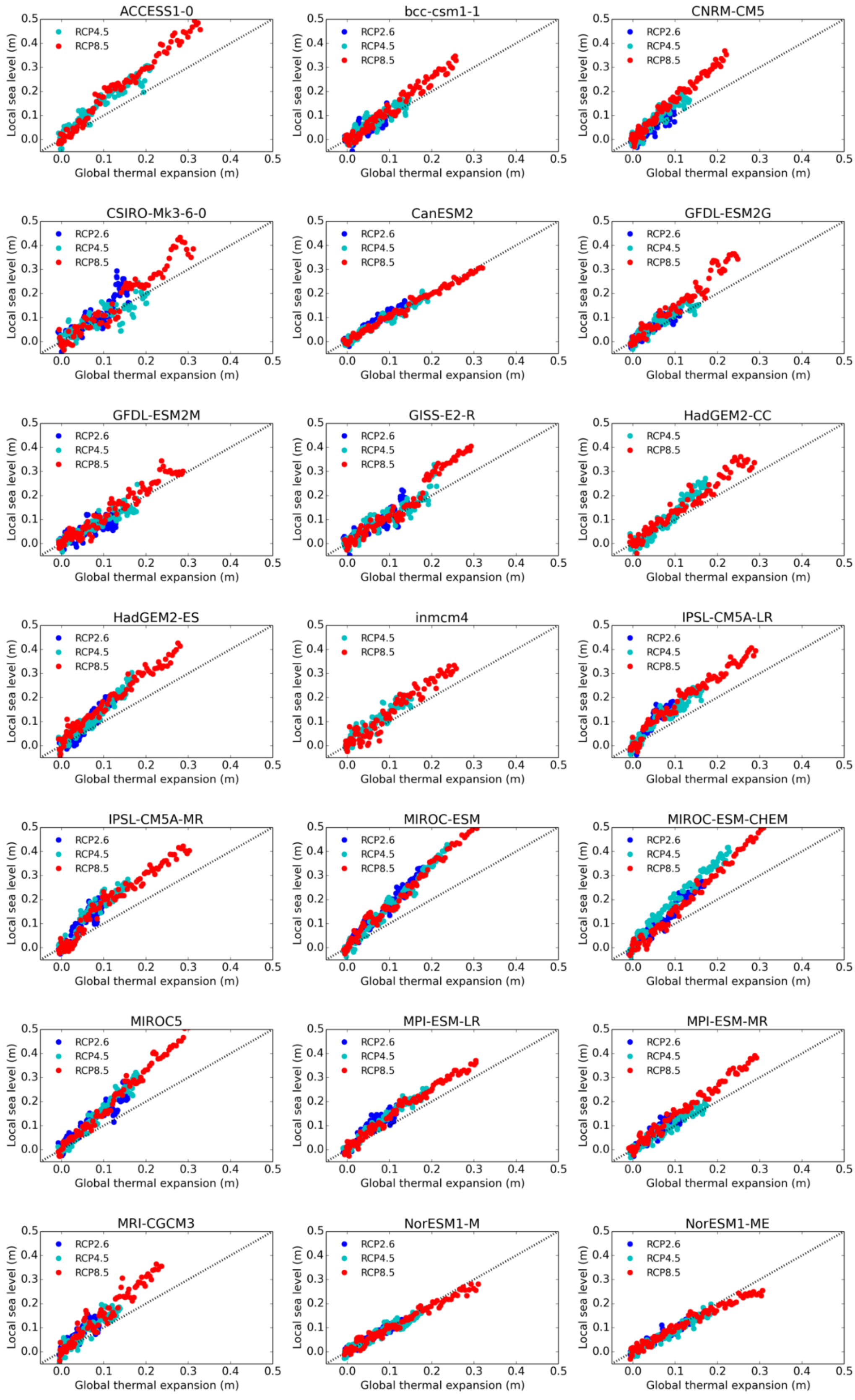

Supplementary Fig. 5: Regression plots of the relationship between local sterodynamic sea level and global thermal expansion for Newlyn (South West England) for all CMIP5 models with data available to 2300 under RCP4.5 based on annual mean data. Colours correspond to the periods 2006-2100 (blue), 2100-2200 (green) and 2200-2300 (red). For comparison, the dotted line indicates the 1:1 relationship. Reproduced from the UKCP18 Marine Report[6].

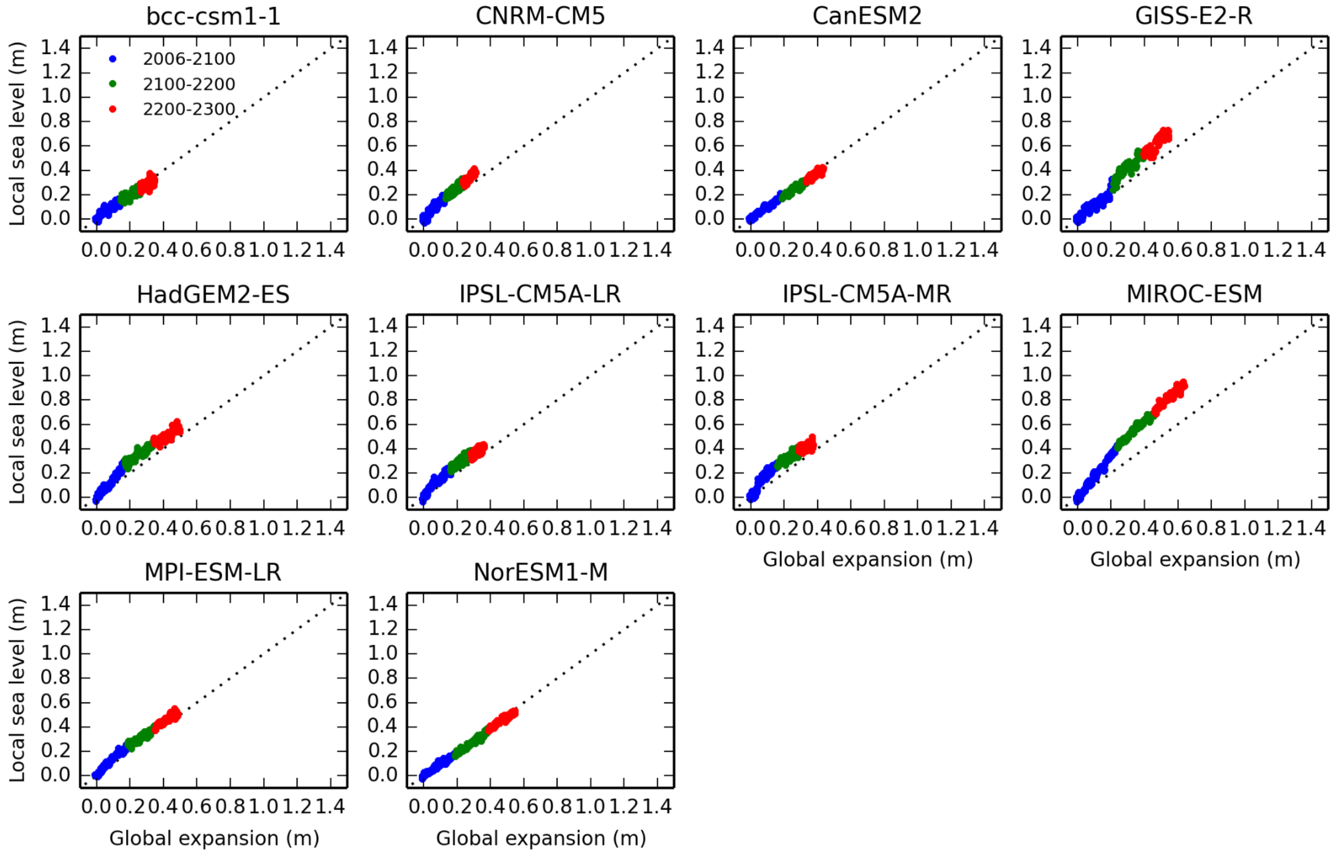

Supplementary Note S3: Flood defence crest height analysis

To assess under what storyline and time horizon coastal flood defences may become overtopped during a 1-in-200-year storm surge event, we have taken defence crest elevations from the Environment Agency Asset Information Management System (AIMS) dataset[9] and assessed the percentage of defences where the modelled water surface elevation is higher than the defence data. We filtered this dataset to remove any defences that were not associated with fluvial or tidal flooding and extracted the maximum modelled water surface elevation along the defence polyline. As the model is an undefended model, there should be some caution in interpreting the exact numbers, which will also be subject to uncertainties introduced through the modelling chain, such as in the DEM or the boundary conditions. We therefore applied an additional filter where if any defence height was exceeded in the present day, we removed it from the analysis so that we could focus on changes in defence overtopping. We note that the Environment Agency AIMS dataset, and hence our analysis, is limited to flood defences in England.

Table S6 gives an indication of when defences could be exceeded under each sea-level rise storylines. It shows that under Storyline B, 21% of defences would be overtopped during a 1-in-200-year storm surge event in 2100. This increases to 51% for Storyline H2. By 2300, these values increase to 64% and >99% of flood defences respectively. However it is reasonable to assume that defences could be locally increased in advance of a flood event (through sandbags for example), or have the design elevation increased in the future. We have therefore computed the same values but for flooding to exceed the defences by 1 and 2 meters. These give a clearer indication of locations where hard adaptation limits may be reached in the future.

Under Storyline B, the percentage of defences that would be overtopped by 1 m or more is 0% for 2100 and 35% for 2300, with 0% and 2% of defences overtopped by 2 m or more. This suggests that under Storyline B, hard adaptation limits will generally not be reached by 2100. However, approximately half of England's coastal flood defences would need investment to increase their defence heights in the subsequent 200 years to maintain the standard of defences, with a small number of defences potentially being beyond hard adaptation limits and requiring alternative approaches such as managed retreat or larger infrastructure investments to be made in the form of new flood defences.

Under Storyline H2, we find by 2100 that 10% of defences would be overtopped by 1 m or more increasing to >99% by 2300. This suggests that if we follow Storyline H2, significant levels of investment

would be required over the coming century. However, hard adaptation limits are unlikely to be reached for the majority of sites, with only 0.3% of locations seeing defences overtopped by 2 m or more by 2100. However, under this storyline, >99% of flood defences become overtopped by greater than 2 m by 2200.

Supplementary Table 8: Percentage of flood defences overtopped in England based on sea-level rise storylines. The percentage of coastal and tidal Environment Agency flood defences that are overtopped during the 1-in-200-year return period event at 2100, 2200 and 2300 for the five storylines described in the main text. All results are reported to 1 decimal place. Any defences modelled as overtopped during the present day are removed from the analysis.

Storyline	Time Horizon	% of coastal and tidal Environment Agency flood defences overtopped			
		0.0m Threshold	0.3m Threshold	1.0m Threshold	2.0m Threshold
Story A	2100	11.8	0.5	0.0	0.0
Story A	2200	39.0	27.1	1.5	0.0
Story A	2300	29.8	15.0	0.5	0.0
Story B	2100	21.0	8.5	0.1	0.0
Story B	2200	58.3	50.1	27.1	0.5
Story B	2300	63.7	55.4	35.3	1.9
Story C	2100	43.5	31.5	1.8	0.1
Story C	2200	82.4	79.2	68.1	45.7
Story C	2300	91.8	89.4	84.9	75.3
Story H1	2100	42.6	30.3	1.8	0.1
Story H1	2200	96.9	96.3	95.2	90.3
Story H1	2300	99.5	99.4	99.3	99.1
Story H2	2100	51.5	43.2	10.0	0.3
Story H2	2200	99.5	99.5	99.4	99.4
Story H2	2300	99.6	99.6	99.6	99.6
Present Day		0.0	0.0	0.0	0.0

REFERENCES

1. Palmer, M. D. *et al.* A framework for physically consistent storylines of UK future mean sea level rise. *Clim. Change* **177**, 106 (2024).
2. Intergovernmental Panel On Climate Change (Ipcc). *Climate Change 2021 – The Physical Science Basis: Working Group I Contribution to the Sixth Assessment Report of the Intergovernmental Panel on Climate Change*. (Cambridge University Press, 2023). doi:10.1017/9781009157896.
3. *National Risk Register 2023*. 192 https://assets.publishing.service.gov.uk/media/64ca1dfe19f5622669f3c1b1/2023_NATIONAL_RISK_REGISTER_NRR.pdf.
4. *Explaining Uncertainty in UK Intelligence Assessment*. https://www.gov.uk/government/publications/explaining-uncertainty-in-uk-intelligence-assessment/explaining-uncertainty-in-uk-intelligence-assessment.
5. DeConto, R. M. & Pollard, D. Contribution of Antarctica to past and future sea-level rise. *Nature* **531**, 591–597 (2016).
6. Palmer, M. *et al.* UKCP18 marine report. (2018).
7. Tinker, J. *et al.* Dynamical downscaling of unforced interannual sea-level variability in the North-West European shelf seas. *Clim. Dyn.* **55**, 2207–2236 (2020).
8. Hermans, T. H. J. *et al.* Improving sea-level projections on the Northwestern European shelf using dynamical downscaling. *Clim. Dyn.* **54**, 1987–2011 (2020).
9. *AIMS Spatial Flood Defences (Inc. Standardised Attributes)*. https://www.data.gov.uk/dataset/cc76738e-fc17-49f9-a216-977c61858dda/aims-spatial-flood-defences-inc-standardised-attributes (2025).
