# Supplementary material for: Quantifying UK coastal flood exposure under future sea-level rise to 2300
Source: Nat Commun. 2026 Jul 21;17:5882. doi: 10.1038/s41467-026-74982-1 (PMC13389417; doi:10.1038/s41467-026-74982-1)
Supplement: Supplementary file 3 — Transparent Peer Review file [file 41467_2026_74982_MOESM3_ESM.pdf]

nature portfolio

Peer Review File

Quantifying UK coastal flood exposure under future sea-level rise to 2300

Corresponding Author: Dr Matthew Palmer

This file contains all reviewer reports in order by version, followed by all author rebuttals in order by version.

Version 0:

Reviewer comments:

Reviewer #1

(Remarks to the Author)
This manuscript presents coastal impacts of sea-level rise and flooding for the 3 coming centuries, assuming sea-level scenarios that have been carefully designed in previous studies. A strength of this study is clearly the use a highly precise digital elevation model (Lidar) and a physical model that represents coastal and estuarine flooding more accurately than most previous assessments at that scale (UK). I am not aware of such a precise assessment at that scale. The authors explain that these results imply that for high sea level storylines, there is no choice but relocation. Due to the effort to implement a more robust coastal flood modeling framework at the scale of an entiere country and due to the significance of the implications, I think that the paper is relevant to Nature Communication.

Major comments

1. coastal adaptation assumptions are not explicitely presented in this manuscript. To understand exactly what are the assumptions regarding protection and adaptation, the reader needs to go to the method section. There, refering to the commond terminology on adaptation scenarios (e.g. Eurosion, 2004, IPCC/SROCC, 2019), I understand that the adaptation scenario considered here is a "do nothing scenario", in which the coastal defenses are assumed to be removed, coastal bathymetries and topographies are unchanged over 100 to 300 years (no erosion/sedimentation). This makes sense as a baseline scenario, but I believe it should be made clear very early in the manuscript so that the reader understands the implications, potential and limitations of selecting this particular adaptation scenario.
For example, this scenario raises the following questions that are not explicitely explained in the manuscript:
- This scenario is one among many other adaptation scenarios, including protection, relocation and implementing nature based solutions, partial relocation (protecting further inland to recreate a larger intertidal area. The do "nothing scenario" is also not the scenario that we see on the ground according to IPCC SROCC, in practice people often protect or accommodate, or they organise a partial relocation. Therefore my recommendation is to be more clear about what we learn from this particular scenario, assuming "do nothing adapatation" for the other scenarios.
- a major implication of this work is that at some point, adaptation limits would probably be reached. However, the discussion on adaptation limits is supported mostly by a 2009 paper about the limits to engineering adaptation in the Thames river estuary, and it is unclear whether it includes the possibility of building a new barrier further offshore. The manuscript would be much stronger if it was able to demonstrate that, if the adaptation scenario consists in protecting coastlines against sea level rise, then, hard adaptation limits will be reached at some point and for some scenarios. This aspect should be strengthenned to support the important (and I believe very true) statement that "[High SLR storylines] would imply the need for large-scale movement of populations and settlements away from the coast in the coming centuries" in the abstract.
- the assumptions made for adapatation and protection also raise questions about coastal flood modeling, which should be more explicit: for high sea level scenarios and if defenses are removed, some areas will be permanently flooded or flooded at high tides, implying coastline changes, which I am not sure are included in the modelling framework, and bathymetric changes (erosion/sedimentation) which are not considered. This will in turn modify extreme water levels during storms. Again, these assumptions make sense, but I believe that they need to be better explained.

2. the manuscript raises questions about climate change mitigation that are very similar to those mentioned in Clark et al 2016. I suggest that this study is considered in the introduction, and overall, I suggest not to limit the discussion on the implications of the results to adaptation needs or options, but also to the question of integenerational justice and mitigation

3. Unlike several previous studies, this study assumes no change in future populations.This is a modelling choice which I think should be explicitely acknowledged, also reminding that population at risks will change in the future (see e.g. Merkens et al 2016).

Suggested reference

Clark, P. et al. 2016 https://doi.org/10.1038/nclimate2923

Merkens et al. 2016 https://doi.org/10.1016/j.gloplacha.2016.08.009

Other minor to moderate comments

- Figure 1 - a: all sea-level rise scenarios present an inflexion after 2100 (in other words, they anticipate that sea level rise will slow down at some point between 2100 and 2200. I understand this is due to the underlying data and assumptions, not the work of the authors, but can they comment about this issue: can this be related to any physical processes or is it a spurious effect due e.g. to the reduced number of models used to design the storylines?
- Line 167-168 - this sentence explains why the 1:200 storm was selected here, and it comes a bit late. Suggest to consider having this justification earlier in the manuscript.
- Line 175-176: I suggest to explain more clearly what "impacted" means in the context of ports for a SLR scnearios above 0.5m (necessity to implement some adaptation options? reaching adaptation limits? Others?)
- Line 178 : same comment for airports
- Line 302-305: I am not sure to understand this approach: does it mean that the regional variability of sterodynamic sea level changes is fixed, whatever the time horizon, or does it mean that the projected patterns of sterodynamic sea level changes are amplified over time after 2100? Can the authors justify this choice, if not in the method section, in the supplementary material?
- Line 338: I don't undersdtand this sentence: does it mean that the omission of flood defenses is considered a second order factor here? This is likely to be true for large amounts of sea-level rise, but for small amounts, this needs to be demonstrated.

- Extended data tables and Supplementary data: The results are presented with a lot of significant digits, giving a false sense of their actual precision. I suggest to round these results or add a note about precision and accuracy.

Reviewer #2

(Remarks to the Author)
The main objective of this paper is to develop and examine a well-founded set of potential coastal flood exposure scenarios for the UK over the extended period until 2300. The study builds upon two previously developed and published frameworks: one for regional sea-level rise (SLR) storyline construction (Palmer et al. 2024), and another for nationwide estimation of flood-related risks using a high-resolution, hydrodynamically based approach (Bates et al. 2023). The novelty of this work lies in the generation of high-resolution large-scale projections of areas at risk of flooding for time horizons extending beyond the widely accepted and commonly analyzed next century, including, among others, low-probability but high-impact scenarios associated with future ice-sheet instability.

The paper raises and substantiates important questions about the prolonged nature of SLR, the increasing uncertainties in the more distant future due to lacking understanding of underlying physical processes, and the potential implications and shortcomings of short-term planning on the example of the present-day infrastructure and exposure for the UK. Additionally, a notable aspect of physical limitations to the defense facility upgrades under very extreme SLR scenarios was emphasized. The paper is clearly structured, includes relevant references and employs a technical methodology that has been established and validated in previous publications.

Here are some specific comments:
1. While the flood-affected areas and population for the 2300 comprise new information and cannot be cross-validated, it would be useful to have some brief comparison, discussion and references on the previous studies about the change in population exposure due to SLR at least for 2100, where enough studies are available (e.g. Vousdoukas et al. 2020, Nature Comm.).
2. The absence of defense facilities in the proposed model is discussed and justified. Thus, the results for the affected population provide an upper and improbable limit of possible flood impact. However, it is not mentioned in the abstract, and the numbers could be misleadingly interpreted as risks under the current state of protection.
3. The study is focused on the impact of SLR. This is not the only, although probably the most substantial, reason for changes in flood exposure. However, for the sake of completeness, it would be helpful to mention potential changes in e.g. storm surge extremes and whether they could be neglected in this context. Another unconsidered aspect is the change in the future population rate and spatial distribution (L. 213), which could alter the results significantly. It is clearly not the purpose of this study, but the absence of population scenarios could be mentioned and justified either in Introduction or Discussion section.

And some questions to the Methodology section:
L 297: “storyline is selected to optimize the fit against individual contributions from…” – not quite clear what was done here. The Monte Carlo realization, most consistent with the selected GMSL percentile for different storylines, was selected. Were there also Monte Carlo simulations for each component (ice sheet, thermosteric contribution, etc.) or which contributions are

meant here? Please, either reformulate or provide relevant references.

L 302: The regression coefficients (global thermosteric to local sterodynamic) were adopted from UKCP18. As far as I know, only standard RCP/SSP scenarios were considered there. What are the coefficients for the H1, H2 storylines and where do they originate from?

L 321: “coastal boundary conditions taken from an Environment Agency extreme value frequency analysis” – are these data used for model validation/calibration? Because it looks like for the Storylines the coastal boundary conditions should be taken from scenarios directly (SLR+extremes).

L 330-342: The procedure is not entirely clear. Does “hydrodynamics” here refer to the inundation model or to the offshore model to generate coastal boundary conditions? What is meant by “event” – is it a typical surge event with a maximum surge level corresponding to 1/200-year return value? Alternatively, does it refer to the 1/200-year return value from the frequency analysis mentioned in L321-L322, which is linearly superimposed on the SLR Storylines? At what stage is the tidal component included - within the offshore hydrodynamic simulation, if there is any (to capture SLR-tide-surge interactions) or linearly added to (SLR + 1/200-yr extreme) value? Does the shape of the surge curve (duration) play any role for the inundation model, or are the duration and volume of water treated as instantaneous and unlimited?
Clarifying the terms “event” and “hydrodynamic simulation” would likely make the methodology easier to follow.

L 334: seems to be too specific and superfluous for the readers outside the UK

Reviewer #3

(Remarks to the Author)
In this study, the authors apply a coastal inundation model to compute the present and future area and population exposure to the 1-in-200 year flood event in the UK under different storylines of future sea-level change. I found the presented results interesting and the manuscript succinct and well written. The flood exposure changes that the authors project clearly warrant intensified mitigation and adaptation action.

Having said that, most of the rather general conclusions drawn by the authors appear to be based on the sea-level storylines themselves instead of on the inundation modeling. Furthermore, the novelty of the study is, in essence, a relatively straightforward translation of existing sea-level rise scenarios to exposure changes using an existing inundation model and several simplifying assumptions. Multiple opportunities for scientific advances regarding the exposure modeling were not capitalized on. I therefore think that the manuscript could be revised to increase its currently somewhat limited contribution to the existing literature. I hope my comments below will be helpful to do so.

Main comments
1. I find a large part of the Discussion (and conclusions) section too general. In my opinion, most of the key conclusions in L229-270 could have already been drawn without the inundation and exposure analysis. For instance, the conclusion that “this combination of sea level rise allowances and residual uncertainty allowances should therefore likely accommodate the values of sea-level rise for the UK across all storylines that have peak values of about 1.5 m” (L237-239) is based purely on the sea-level storylines and system knowledge, not on the exposure changes. The same argument applies to most of L254-270. While the points that the authors raise here are clearly valid and relevant, they do not relate to the results of this study directly enough and overlap with the main messages of previous studies as well (e.g., Palmer et al., 2024; Turner et al., 2025, Slangen et al., 2022, etc.). I would therefore encourage the authors to provide a more detailed discussion of their inundation/exposure modeling results, clarify what specifically these results mean for mitigation and adaptation planning in the UK, and include a discussion of how their results compare to previous flood exposure studies, which is currently lacking.

2. L17-18, L266-268, L275-276: the authors conclude from their analysis of flood exposure that under storylines with high sea-level rise, ‘maintaining current coastlines will no longer be economically viable for most of the UK’ and that this ‘necessitates large-scale movement of settlements, population and infrastructure’. These conclusions are not directly supported by the analysis. Namely, the authors only compute changes in hazards and exposure to the 1-in-200 year event, not in overall impact and risk. Furthermore, they do not provide any cost/benefit analysis of different adaptation strategies (as in, e.g., Tiggeloven et al., 2020, Ballesteros et al., 2025). Finally, as seen in countries already strongly impacted by sea-level rise, the lack of economic viability of coastal protection does not necessarily mean that migration and retreat is needed or will follow (e.g., Esteban et al., 2019). I think these aspects need to be incorporated.

3. The authors make a lot of simplifications in their modeling approach that, in my view, detract from the scientific value of their study:
a. L302-304: existing analyses with both global and regional climate models suggest that (1) the pattern of sterodynamic sea-level change around the UK is not spatially uniform (e.g., Fox-Kemper et al., 2019; Hermans et al., 2020) and that (2) the relationship between global mean thermal expansion and regional to local ocean dynamic sea-level change is not necessarily linear, especially not on the longer timescales considered in this manuscript (Malagon-Santos et al., 2023, 2025). It would help to address these aspects or at least justify their neglection.

b. The authors ignore socioeconomic developments which, especially at multi-centennial timescales, can also be an important driver of changes in exposure (e.g., Hinkel et al., 2021; Lincke et al., 2022; Ballesteros et al., 2025). It could be insightful to include scenarios of, for instance, population changes in the exposure modeling, or at least their omission

should be more elaborately discussed in the context of similar studies that do include such changes.

c. It is unclear if/how the authors account for the uncertainty in the height of the 1-in-200 year return event, which could be comparable to the uncertainty of mean sea-level rise (Wahl et al., 2017). Including this uncertainty could be a relatively straightforward step to increase the novelty of the study.

A related point are dynamic changes in tides, waves and surges, which could substantially affect the changes in the exposure as well. How valid is it to ignore these changes on these long timescales? And could it be useful to incorporate storylines of such changes as well based on previous work by the authors (e.g., https://iopscience.iop.org/article/10.1088/2515-7620/ad3368)?

d. Adaptation measures (present or future) were not incorporated. The authors' motivation that this is a 'second-order factor' (L338) is clearly incorrect: in fact, it is one of the most important factors in assessments of coastal flood risk, if not *THE* most important factor (Hinkel et al., 2021). I understand that including current and future adaptation measures involves substantial additional uncertainties, but assessing different adaptation scenarios does provide useful insights (e.g., Tiggeloven et al., 2020; Ballesteros et al., 2025) and in my view, the authors need to discuss this caveat of their work more prominently in the main text.

4. Apologies if I missed it, but I cannot find any code or data availability statement. This would render the study irreproducible and does not comply with FAIR principles.

Other Comments
General: None of the URLs referred to in the paper worked for me, even though some of these references contain information key to the study. I would recommend the authors to appropriately cite these references, including access dates, and to make sure this information will remain accessible in the future.

Figure 1: Different scales are used for each map, which is somewhat confusing since the same colormaps are used. Please consider if a single logarithmic scale could be helpful.

L112-L159: A substantial part of the Results section introduces results of a previous study by the authors (Palmer et al., 2024, ref. 9), not results of the present study, which I found misleading. While I understand this is important background information, the same information is also presented in the Online Methods. Perhaps the paragraphs in the main text could therefore be condensed to a few sentences, and a table or subfigure that briefly summarizes the characteristics of each storyline could be added to Figure 1?

L133-134: The bracket notation is slightly confusing – please consider spelling this out for clarity.

L166-167: Since the authors do not consider flood defenses in their analysis, it is unclear to the reader what this statement is based on. Would it be helpful to add a few examples?

L297: "optimize the fit against" I didn't understand what this means, sorry. Could you please clarify?

L321-L334: these lines discuss the extreme sea-level analysis used as input to the inundation model. Besides that the URL referred to does not seem to work, this is methodological information that in my opinion should be included in the article itself, especially given the central role of the 1-in-200 year return event in this study.

L341-342: It was not clear to me what this refers to, does this mean that surges and tides are linearly combined and if so, how valid is that assumption?

References other than already cited:
Ballesteros et al. (2025) https://doi.org/10.3389/fmars.2025.1505633
Esteban et al. (2019) https://doi.org/10.1016/j.ocecoaman.2018.10.031
Hermans et al. (2020) https://doi.org/10.1007/s00382-019-05104-5
Hinkel et al. (2021) https://doi.org/10.1029/2020EF001882
Lincke et al. (2022) https://doi.org/10.1029/2021EF002584
Tiggeloven et al. (2020) https://doi.org/10.5194/nhess-20-1025-2020
Turner et al. (2023) https://doi.org/10.1029/2023EF003550
Malagon-Santos et al. (2023) https://doi.org/10.5194/os-19-499-2023
Malagon-Santos et al. (2025) https://doi.org/10.1088/1748-9326/ade906
Slangen et al. (2022) https://doi.org/10.1029/2021EF002576
Wahl et al. (2017) https://doi.org/10.1038/ncomms16075

Version 1:

Reviewer comments:

Reviewer #1

(Remarks to the Author)
I thank the authors for responding satisfactorilly to my previous comments. I only have one final minor comment: given the modelling framework used by the Authors to evaluate total water levels at the coast, I am not sure that waves are taken into account. This means that in the final paragraph of the discussion, the defenses would not be overtopped but rather overflown by approximately 1m, which indeed would generate a major flood event. This is because many coastal engineers define overtopping as mean total water levels being below flood defenses, but waves generating flooding beyond coastal defenses. I just recommend to check this terminology point and adjust.

I hope it is useful

Gonéri le Cozannet, BRGM, 16th March 2026

(Remarks on code availability)

Reviewer #2

(Remarks to the Author)
The main questions are clarified satisfactorily.

The second draft benefits from an additional consideration of coastal defense crest heights and corresponding statistics on overtopping. This illustrates SLR potential impacts, especially for high-end storylines, in a more comprehensive and relatable way.

Minor comment:
Supplement Note S2 and related reply to Rev.1: it looks like here the explanation about the space-time invariant coefficients for sterodynamic regional sea-level rise is relevant only for the thermosteric part of GMSL rise (i) and not for the entire GMSL/RMSL relation, as also described in the main manuscript. Otherwise, it would contradict the regional spatial variability presented in Fig.1 and following discussion. So, in the present form the title of the Note2 is misleading and should somehow include “thermosteric GMSL” term to underline only partial space-time uniformity.

(Remarks on code availability)
Could't access the page with the message "Permission required"

Reviewer #4

(Remarks to the Author)
This paper extends earlier analyses of the implications of sea-level rise for the UK. It links the sea-level rise outputs from IPCC AR6 to 2300 and also considered the national coastal flood implications to 2300. This compares to earlier analyses which only extended to 2100.

The analysis is credible and interesting in a technical sense. However, with any such analysis there are inevitably -- so what? -- or --what if? -- kinds of questions. Below I pose the key questions that spring to mind. I also identify a few smaller points in the manuscript.

Once these are addressed, I would like to see the authors responses and expect to be happy to see this paper published.

1. What is a meaningful reasonable worse case? The high-end scenarios of IPCC AR6 assume high emissions linked to the RCP8.5 emissions scenario. These are considered rather unlikely and yet are often analysed like they are business-as-usual (e.g. Hausfather and Peters, 2020). So there is an unlikely emissions scenario driving the H1 and H2 storylines. And these outcomes are associated with one model available to the AR6 or one line of evidence. In contrast, the earlier H++ scenario was associated with multiple lines of evidence --- process models, palaeoclimate analogues, semi-empirical models, etc. all seemed to justify a number around 2 m in 2100. Hence, when you come to a reasonable worst case as you define it, which is a “challenging manifestation of the scenario after highly implausible scenarios are excluded” what are the highly implausible scenarios you have excluded? It might be argued that you have taken the biggest number you have got? So why not consider the H2 as the reasonable worst case? Note that another line of evidence can support it – van de Wal et al (2022) -- which is independent of the IPCC AR6 analysis. At the end of the day, this is down to expert judgement but a little more justification of why you pick H1 over H2 would be good including acknowledging issues around emissions. And is it possible to have two reasonable worst cases – the H1 and H2 cases? So please review the paper in the light of the above points and improve your justification for your selection, including acknowledging RCP8.5 is an unlikely scenario and the high end scenarios are exploratory.

2. What is the purpose of the analysis? It states it is to inform adaptation planning although first and foremost it informs on exposure and risk. In terms of the interpretation of exposure and risk, over the next century or so it will inform adaptation. But beyond this it is informing wider climate policy as we would like to avoid an H1 and H2 world if at all possible. This gives more impetus to climate mitigation although adaptation is an inevitable part of the mix with the UK already having large coastal floodplains. It might also raise thoughts about geoengineering interventions in Antarctica (e.g., Leane and McGee,

2020; Moore et al., 2021). I am not a fan of these proposals, but they are out there and if you raise catastrophic changes these mega interventions will also be raised. So please review the stated purpose of the paper and how it realistically informs policy throughout the manuscript.

3. For the H2 scenario in 2300 why do you not also consider inundation – i.e. those areas that would be below normal high tides so underwater almost everyday without defences. The analysis talks about flooding – but for much of the flooded area, the flood would never go away. So please make sure that the manuscript conveys this important point.

4. The Environment Agency only cover England and this should be indicated to the reader when this is used (e.g. Supplemental Table S8).

Smaller points
L31 – 'primary threat' – there is no other threat of multi-metre SLR except the two big ice sheets so this needs rewording.
L54-57 – this statement is true but so what? --- it is comparing a 2100 projection to 2300 projections– think about the point and rephrase – big rises are possible as we extend timescales beyond a century?
L91-92 1 in 200 standard is the typical design standard for urban areas – less densely populated areas would receive lower protection standards and maybe nothing depending on what is exposed.
L121-122 'British and Irish Isles' – puzzled by this. If this is really important to you maybe say 'Britian and Ireland' as this makes sense to me.
L136 Is storyline H2 'informing future coastal adaptation planning' or 'more widely emissions and adaptation planning'. I think the goal here is much more to avoid this outcome with climate mitigation rather than prepare an adaptation plan.
Line 157-160 Ports are probably incrementally upgrading already? Port design manuals already include (small) allowances for sea-level rise.

References

Hausfather, Z. and Peters, G.P., 2020. Emissions–the 'business as usual' story is misleading. Nature, 577(7792), pp.618-620.
Leane, E. and McGee, J., 2020. Anthropocene Antarctica. Routledge.
Moore, J.C., Mettiäinen, I., Wolovick, M., Zhao, L., Gladstone, R., Chen, Y., Kirchner, S. and Koivurova, T., 2021. Targeted geoengineering: local interventions with global implications. Global Policy, 12, pp.108-118.

(Remarks on code availability)

Version 2:

Reviewer comments:

Reviewer #4

(Remarks to the Author)
Thank you for your thoughtful responses to my review. I have no further remarks and I am happy to see the paper published.

Open Access This Peer Review File is licensed under a Creative Commons Attribution 4.0 International License, which permits use, sharing, adaptation, distribution and reproduction in any medium or format, as long as you give appropriate credit to the original author(s) and the source, provide a link to the Creative Commons license, and indicate if changes were made.
In cases where reviewers are anonymous, credit should be given to 'Anonymous Referee' and the source.
The images or other third party material in this Peer Review File are included in the article's Creative Commons license, unless indicated otherwise in a credit line to the material. If material is not included in the article's Creative Commons license and your intended use is not permitted by statutory regulation or exceeds the permitted use, you will need to obtain permission directly from the copyright holder.
To view a copy of this license, visit https://creativecommons.org/licenses/by/4.0/

RESPONSE TO REVIEWER COMMENTS

We would like to thank the three reviewers for their thoughtful and constructive comments on our manuscript. Before going into a point-by-point response to their comments, we briefly present an overview of the substantive revisions we have made. Our responses to reviewer comments appear in blue with text lifted from the revised manuscript materials shown in quotes and *italic* font.

1. Several reviewers asked us to be more explicit about justification for methodological assumptions and to more clearly state the limitations of our study. We have substantively revised the introductory text in the main manuscript, the Methods and the Supplementary Information in light of these comments.

2. The revised materials include a new flood defence crest height analysis to support the main findings of the paper. This analysis explores the time-horizons on which present-day defences might be over-topped due to future sea-level rise. Details can be found in Note S3 and Table S6 of the revised Supplementary Materials.

3. We have strengthened the discussion on population exposure (including a comparison at 2100 with a previous study) and implications for adaptation planning, including expanded discussion and evidence for reaching adaptation limits (see point above).

4. We have revised Figures 1 and Supplementary Figures 1 and 2 in light of review comments.

5. We have introduced a new Table 1 in the main manuscript that summarises the narrative elements of the sea-level rise storylines used in this study.

6. We have made extensive changes throughout the manuscript text in light of reviewer comments (see tracked changes file).

PLEASE NOTE: The original manuscript was prepared for *Nature Climate Change*, which has different section headings/format than *Nature Communications*. To aid the reviewers, the track changes version of the manuscript preserves the formatting of the original submission (i.e., based on *Nature Climate Change*), including the Supplementary Information. The clean version of the manuscript follows the formatting of *Nature Communications*. This means that all Extended Data figures and tables from the original submission now appear in the revised Supplementary Information for the clean version of the manuscript.

Extended Data Figure 1 => Supplementary Figure 1
Extended Data Figure 2 => Supplementary Figure 2
Extended Data Figure 3 => Supplementary Figure 3

Extended Data Table 1 => Supplementary Table 1
Extended Data Table 2 => Supplementary Table 2
Extended Data Table 3 => Supplementary Table 3
Extended Data Table 4 => Supplementary Table 4

Reviewer #1 (Remarks to the Author):

This manuscript presents coastal impacts of sea-level rise and flooding for the 3 coming centuries, assuming sea-level scenarios that have been carefully designed in previous studies. A strength of this study is clearly the use a highly precise digital elevation model (Lidar) and a physical model that represents coastal and estuarine flooding more accurately than most previous assessments at that scale (UK). I am not aware of such a precise assessment at that scale. The authors explain that these results imply that for high sea level storylines, there is no choice but relocation. Due to the effort to implement a more robust coastal flood modeling framework at the scale of an entiere country and due to the significance of the implications, I think that the paper is relevant to Nature Communication.

Major comments

1. coastal adaptation assumptions are not explicitely presented in this manuscript. To understand exactly what are the assumptions regarding protection and adaptation, the reader needs to go to the method section. There, refering to the commond terminology on adaptation scenarios (e.g. Eurosion, 2004, IPCC/SROCC, 2019), I understand that the adaptation scenario considered here is a "do nothing scenario", in which the coastal defenses are assumed to be removed, coastal bathymetries and topographies are unchanged over 100 to 300 years (no erosion/sedimentation). This makes sense as a baseline scenario, but I believe it should be made clear very early in the manuscript so that the reader understands the implications, potential and limitations of selecting this particular adaptation scenario.
For example, this scenario raises the following questions that are not explicitely explained in the manuscript:
- This scenario is one among many other adaptation scenarios, including protection, relocation and implementing nature based solutions, partial relocation (protecting further inland to recreate a larger intertidal area. The do "nothing scenario" is also not the scenario that we see on the ground according to IPCC SROCC, in practice people often protect or accommodate, or they organise a partial relocation. Therefore my recommendation is to be more clear about what we learn from this particular scenario, assuming "do nothing adapatation" for the other scenarios.
- a major implication of this work is that at some point, adaptation limits would probably be reached. However, the discussion on adaptation limits is supported mostly by a 2009 paper about the limits to engineering adaptation in the Thames river estuary, and it is unclear whether it includes the possibility of building a new barrier further offshore. The manuscript would be much stronger if it was able to demonstrate that, if the adaptation

scenario consists in protecting coastlines against sea level rise, then, hard adaptation limits will be reached at some point and for some scenarios. This aspect should be strengthenned to support the important (and I believe very true) statement that "[High SLR storylines] would imply the need for large-scale movement of populations and settlements away from the coast in the coming centuries" in the abstract.
- the assumptions made for adapatation and protection also raise questions about coastal flood modeling, which should be more explicit: for high sea level scenarios and if defenses are removed, some areas will be permanently flooded or flooded at high tides, implying coastline changes, which I am not sure are included in the modelling framework, and bathymetric changes (erosion/sedimentation) which are not considered. This will in turn modify extreme water levels during storms. Again, these assumptions make sense, but I believe that they need to be better explained.

We agree with the reviewer that we could be more explicit earlier on about the defence scenario considered and the implications of this. We have now explicitly referred to the scenario as being undefended in the abstract so that readers immediately understand that exposure estimates are based on undefended model scenarios. We also agree that this should be explicitly discussed further in the main text to make the reader aware of both the implications but also the reasonings and consequences for this modelling choice. This has now been expanded in the methods section:

"*We use model simulations without flood defences for several reasons. Firstly, it allows us to identify: (i) new populations that could become exposed to flooding in the absence or failure of flood defences; (ii) infrastructure or land that could become exposed to floods which could help inform decisions on whether areas require future investment in flood protection infrastructure; and (iii) locations that could be repurposed for relocated communities to inhabit. Secondly, there are no datasets that project flood defence standards out to 2300, and the level of investment in defences would also likely vary depending on the climate scenario followed. Furthermore, if we assume defences remain at their current standards of protection, we would learn very little about changing flood risk except in undefended and rural areas. Therefore we feel that the undefended scenario provides a more complete picture of potential exposure to floods both in the present day and the future."*

We have also added some additional text into the discussion that builds upon this:

"The results have identified many instances of major infrastructure and nationally significant sites moving into flood risk areas as we move to scenarios beyond 2100. This is in addition to the greater than five-fold increases in populations that could become at risk of flood exposure under the H2 scenario. Increased exposure to floods is identified across all scenarios, and it is therefore inevitable that a combination of additional flood defence spending and managed retreat will be necessary to mitigate against flood risk beyond 2100. The fact that we have already identified existing infrastructure that could become exposed to flooding in the future raises the important question as to whether coastal flood risk estimations beyond 2100 should be regularly incorporated into future major

infrastructure development and urban expansion planning. Such a move could help avoid costly future interventions should some of the more extreme future SLR scenarios be realised and help identify locations that may be better placed to avoid future flood risk on these timescales."

On the point of hard infrastructure limits being met, the original manuscript included commentary on the Thames barrier being overwhelmed with 5m SLR, however we agree the more general point could be strengthened. To do this, we have undertaken and included additional analysis, comparing our outputs to all known UK flood defence crest elevations to give an indication of when these limits may be met.

We have taken defence crest elevations from the Environment Agency AIMS dataset (Environment Agency, 2025) and calculated the percentage of defences where the modelled water surface elevation is higher than the defence data for each scenario and time horizon. We have described this in a new section in the Supplementary Information (Note S3: Flood defence crest height analysis) and referred to this in the main text. This has provided important information on the time horizons and scenarios at which hard adaptation limits are likely to have been met. We have included the following text and table within the SI:

"To assess under what storyline and time horizon coastal flood defences may become overtopped during a 1-in-200-year storm surge event, we have taken defence crest elevations from the Environment Agency Asset Information Management System (AIMS) dataset (Environment Agency, 2025) and assessed the percentage of defences where the modelled water surface elevation is higher than the defence data. We filtered this dataset to remove any defences that were not associated with fluvial or tidal flooding and extracted the maximum modelled water surface elevation along the defence polyline. As the model is an undefended model, there should be some caution in interpreting the exact numbers, which will also be subject to uncertainties introduced through the modelling chain, such as in the DEM or the boundary conditions. We therefore applied an additional filter where if any defence height was exceeded in the present day, we removed it from the analysis so that we could focus on changes in defence overtopping."

Table S6 gives an indication of when defences could be exceeded under each sea-level rise storyline. It shows that under storyline B, 21% of defences would be overtopped during a 1-in-200-year storm surge event in 2100. This increases to 51% for Story H2. By 2300, these values increase to 64% and >99% of flood defences, respectively. However it is reasonable to assume that defences could be locally increased in advance of a flood event (through sandbags for example), or have the design elevation increased in the future. We have therefore computed the same values but for flooding to exceed the defences by 1 and 2 meters. These give a clearer indication of locations where hard adaptation limits may be reached in the future.

Under storyline B, the percentage of defences that would be overtopped by 1 m or more is 0% for 2100 and 35% for 2300, with 0% and 2% of defences overtopped by 2 m or more.

This suggests that under storyline B, hard adaptation limits will generally not be reached by 2100. However, approximately half of England's coastal flood defences would need investment to increase their defence heights in the subsequent 200 years to maintain the standard of defences, with a small number of defences potentially being beyond hard adaptation limits and requiring alternative approaches such as managed retreat or larger infrastructure investments to be made in the form of new flood defences.

Under storyline H2, we find by 2100 that 10% of defences would be overtopped by 1 m or more increasing to >99% by 2300. This suggests that if we follow Storyline H2, significant levels of investment would be required over the coming century. However, hard adaptation limits are unlikely to be reached for the majority of sites, with only 0.3% of locations seeing defences overtopped by 2 m or more by 2100. However, under this storyline, >99% of flood defences become overtopped by greater than 2 m by 2200."

Table S6: The percentage of coastal and tidal Environment Agency flood defences that are overtopped during the 1-in-200-year return period event at 2100, 2200 and 2300 for the five storylines described in the main text. All results are reported to 1 decimal place. Any defences modelled as overtopped during the present day are removed from the analysis.

Storyline	Time Horizon	% of coastal and tidal Environment Agency flood defences overtopped			
		0.0m Threshold	0.3m Threshold	1.0m Threshold	2.0m Threshold
Story A	2100	11.8	0.5	0.0	0.0
Story A	2200	39.0	27.1	1.5	0.0
Story A	2300	29.8	15.0	0.5	0.0
Story B	2100	21.0	8.5	0.1	0.0
Story B	2200	58.3	50.1	27.1	0.5
Story B	2300	63.7	55.4	35.3	1.9
Story C	2100	43.5	31.5	1.8	0.1
Story C	2200	82.4	79.2	68.1	45.7
Story C	2300	91.8	89.4	84.9	75.3
Story H1	2100	42.6	30.3	1.8	0.1
Story H1	2200	96.9	96.3	95.2	90.3
Story H1	2300	99.5	99.4	99.3	99.1
Story H2	2100	51.5	43.2	10.0	0.3
Story H2	2200	99.5	99.5	99.4	99.4

Story H2	2300	99.6	99.6	99.6	99.6
Present Day		0.0	0.0	0.0	0.0

We have also added some additional analysis in the results and discussion sections of the paper to build on this and comment on the implications. For example in the discussion we have included the following two additional sections text:

"Under Storyline B it is unlikely that hard flood defence adaptation limits will be reached by 2100 (Table S6). This is also the case for the majority of defences for Storyline H2, although significant levels of investment would be required to increase the height of defences over the coming century (Table S6).

Under Storyline B, approximately half of England's coastal flood defences would need investment over the subsequent 200 years to maintain defence standards, with a small number potentially being beyond hard adaptation limits (Table S6). Under storyline H2, >99% of flood defences could already be beyond hard adaptation limits by 2200 (Table S6). The increased population exposure to the 1-in-200-year flood event under the H2 storyline from 3 million to almost 10 million between 2100 and 2200 implies potential for widespread disruption to the economy and society, with challenges compounded by potentially reaching hard adaptation limits (Table 6). This scenario would require high levels of investment for both new and existing flood defences, potential movement of communities away from coastlines and put critical infrastructure and sites of national strategic importance at high risk of disruption."

For the reviewer's final point on assumptions and limitations, we have made these explicit in the methods section, as requested:

"We acknowledge that the modelling approach neglects coastline and bathymetry changes associated with erosion and sedimentation processes, which could further modify extreme coastal water levels through associated tide and surge changes. These aspects are likely to be increasingly important under large degrees of future sea-level rise and warrant further research."

Reference:
Environment Agency 2025. AIMS Spatial Flood Defences (inc. standardised attributes) available at: https://www.data.gov.uk/dataset/cc76738e-fc17-49f9-a216-977c61858dda/aims-spatial-flood-defences-inc-standardised-attributes [last accessed 09/02/2026]

2. the manuscript raises questions about climate change mitigation that are very similar to those mentioned in Clark et al 2016. I suggest that this study is considered in the introduction, and overall, I suggest not to limit the discussion on the implications of the

results to adaptation needs or options, but also to the question of integenerational justice and mitigation

We have added the following sentence in the Introduction:
"Previous studies have highlighted the consequences of climate policy decisions in the next few decades for determining sea-level rise outcomes for centuries and millennia to come, illustrating the importance of early emissions reductions and raising questions about intergenerational justice [Clark et al, 2016; Fox-Kemper et al, 2021; Nauels et al, 2025]."

3. Unlike several previous studies, this study assumes no change in future populations.This is a modelling choice which I think should be explicitely acknowledged, also reminding that population at risks will change in the future (see e.g. Merkens et al 2016).

Thanks - this is a good point. We chose not to include population change because suitably detailed demographic projections for the UK beyond 2100 do not exist. Indeed, as far as we are aware, no such projections exist for any country worldwide. Whilst the UK-SSP project has developed downscaled 1km population projections to 2100 for the five shared socio-economic pathways, these do not incorporate information on future flood risk and therefore allow expansion of population into areas which, in reality, would never be developed. In addition, UK-SSP projections do not cover the period 2100-2300, are not sufficiently granular for a very localised problem such as flooding and do not exactly correspond to the storylines used in this paper, particularly for the H1 and H2 scenarios which are very different to anything in UK-SSP. Therefore, the UK-SSP projections are not appropriate for our study.

Nevertheless, we agree that it is important to explicitly acknowledge this modelling choice and we have therefore added text in the main text (last two paragraphs before Results) to outline our rationale for working with the present day population as a baseline:

"We characterise the coastal flood impacts associated with these storylines given present day population for the UK as a whole and for the UK capital cities of Belfast, Cardiff, London and Edinburgh."

And

"*We work with the present day population as a baseline because currently available UK future projections (Merkle et al, 2022) are: (i) 'blind' to flood risk and therefore allow expansion of population into areas which, in reality, would never be developed; (ii) only available at 1km resolution which is insufficiently granular for a localised problem such as flooding; and (iii) only exist out to 2100 as beyond this uncertainties are simply too great. It should be noted, however, that population is not static and future work should incorporate these effects once appropriate projections become available.*"

Reference:
Merkle, M. et al. Downscaling population and urban land use for socio-economic scenarios in the UK. Reg Environ Change 22, 106 (2022).

Other minor to moderate comments

- Figure 1 - a: all sea-level rise scenarios present an inflexion after 2100 (in other words, they anticipate that sea level rise will slow down at some point between 2100 and 2200. I understand this is due to the underlying data and assumptions, not the work of the authors, but can they comment about this issue: can this be related to any physical processes or is it a spurious effect due e.g. to the reduced number of models used to design the storylines?

Thanks for this suggestion. Yes - this is a physical effect (not spurious) related to the peak in greenhouse gas emissions and associated radiative forcing. We have added the following text in this section:

"The model simulations underlying the storylines are based on assumptions of reducing greenhouse gas emissions from the 2020s (Story A), around 2040 (Story B) or from 2150 (Story C, H1, H2) with a subsequent decrease in the rate of GMSL rise thereafter (Figure 1a; Meinshausen et al, 2011)."

- Line 167-168 - this sentence explains why the 1:200 storm was selected here, and it comes a bit late. Suggest to consider having this justification earlier in the manuscript.

Thanks for this suggestion. We have included this information in the second-last paragraph of the opening section of the manuscript, which now reads as:

"Simulations are based on the 1-in-200-year return period extreme coastal water level event from the UK Environment Agency coastal flood boundary condition dataset (see Online Methods) since this is the typical design standard of UK coastal flood defences."

- Line 175-176: I suggest to explain more clearly what "impacted" means in the context of ports for a SLR scnearios above 0.5m (necessity to implement some adaptation options? reaching adaptation limits? Others?)

By 'impacted' we here mean that ports will be exposed to higher extreme water level events as a result of sea-level rise. Because of this, extreme event frequencies which currently do not cause problems may do so in the future and further protection measures may be required. We should note that our work looks only at changing hazard and exposure, and does not simulate impacts and adaptation measures explicitly. Detailed modelling of impacts to major infrastructure would require vulnerability functions or adaptation plans for specific facilities which currently do not exist (at least in the public domain). Nevertheless, we agree that some further discussion of how specific amounts of

sea-level rise are likely to cause impacts is very useful so we have added this text on line 187:

Line 187: *"Nevertheless, many UK port facilities in southern England, which are necessarily outside of flood defences, will be impacted by a ~0.5 m rise in sea level due to the increase in extreme water levels during coastal surge events and this may require additional protection measures to be adopted (Christodoulou et al, 2019)."*

Reference:

Christodoulou, A., Christidis, P. & Demirel, H. Sea-level rise in ports: a wider focus on impacts. *Marit Econ Logist* 21, 482–496 (2019). https://doi.org/10.1057/s41278-018-0114-z

The main point being made here is simply that, under scenario B, most impacts around the UK coast will be zero or negligible by 2100 because of the climate change allowances already built into the design of present day coastal flood defences. However, there may be a small number of infrastructure facilities, such as ports, where 0.5m of sea-level rise could pose a problem. We have attempted to more quantitatively document these effects by including the new analysis of defence crest elevation and when these are overtopped (see response to comments above).

- Line 178 : same comment for airports

Again, we are not specifically modelling detailed impacts, but simply assuming that if infrastructure lies within the new flood zone and is subject to non-negligible water depths then it will suffer a degree of impact that is material.

- Line 302-305: I am not sure to understand this approach: does it mean that the regional variability of sterodynamic sea level changes is fixed, whatever the time horizon, or does it mean that the projected patterns of sterodynamic sea level changes are amplified over time after 2100? Can the authors justify this choice, if not in the method section, in the supplementary material?

In light of the reviewer's comment, we have revised the text in the main manuscript, which now reads as follows:

"Timeseries of (i) are converted to local sterodynamic sea-level change using a spatially uniform regression coefficient based on the scenario-specific central estimates from UKCP18 corresponding to each scenario. These values are: 1.372 for SSP1-2.6/RCP2.6 (used for Storyline A); 1.390 for SSP2-4.5/RCP4.5 (used for Storyline B) and 1.465 for SSP5-8.5/RCP8.5 (used for Storyline C, H1, H2). All regression coefficients result in a time-invariant amplification of the global signal that is largest for the SSP5-8.5/RCP8.5 scenario. Further justification of this approach is presented in the Supplementary Materials."

We have also included a new section in the Supplementary Materials, as suggested by the reviewer, which reads as follows:

*"**Note S2: Approach to local sterodynamic sea-level rise***

Following the methods presented in UKCP18 (Palmer et al, 2018), a scenario-specific space-and-time invariant regression coefficient is used to estimate the local sterodynamic sea change for each storyline, as described in the main text. The assumption of spatial invariance was originally made in UKCP18 because the underlying CMIP5 global climate model (GCM) simulations have limited horizontal and vertical resolution and are missing key processes, such as tides (e.g., Tinker et al, 2020). Therefore it was concluded that any spatial variations around the UK coast were unlikely to be robust and that an assumption of spatial uniformity was more appropriate (Palmer et al, 2018). Subsequently, Hermans et al (2020) have shown that downscaling of CMIP5 GCM simulations results in essentially spatially uniform patterns of sterodynamic sea-level change round the UK coastline.

Under UKCP18, scenario-specific regression coefficients for each RCP scenario were computed on the basis of a linear fit between global thermosteric sea-level rise and local sterodynamic sea-level rise for CMIP5 model simulations over the period 2007-2100 (Figure S1). The same process was carried out for each CMIP5 GCM grid box adjacent to the UK coastline. Given the assumption of spatial invariance noted above, a central estimate value was used across all available CMIP5 UK coastal grid boxes. It was assumed that these regression coefficients were representative of the time-horizon out to 2300, which is a reasonable first-order approximation as illustrated for the available CMIP5 simulations under the RCP4.5 scenario (Figure S2). Further details are available in Palmer et al (2018).

References:

*Hermans, T.H.J., Tinker, J., Palmer, M.D. et al. Improving sea-level projections on the Northwestern European shelf using dynamical downscaling. Clim Dyn **54**, 1987–2011 (2020). https://doi.org/10.1007/s00382-019-05104-5*

Palmer, M. D., Howard, T., Tinker, J., Lowe, J., Bricheno, L., Calvert, D., Edwards, T., Gregory, J., Harris, G., Krijnen, J., Pickering, M., Roberts, C., Wolf, J. (2018). UKCP18 marine report, 133 pp. Available from https://www.metoffice.gov.uk/pub/data/weather/uk/ukcp18/science-reports/UKCP18-Marine-report.pdf

Tinker, J., Palmer, M.D., Copsey, D. et al. Dynamical downscaling of unforced interannual sea-level variability in the North-West European shelf seas. Clim Dyn 55, 2207–2236 (2020). https://doi.org/10.1007/s00382-020-05378-0"

- Line 338: I don't understand this sentence: does it mean that the omission of flood defenses is considered a second order factor here? This is likely to be true for large amounts of sea-level rise, but for small amounts, this needs to be demonstrated.

Apologies, our phrasing here was unclear. We did not intend that flood defences should be considered a second order factor. Rather, what we meant was that even an overtopped flood defence can still have a minor continuing impact on flood extent compared to the situation where there is no defence present at all. This effect (impact of over-whelmed flood defences on continuing flood propagation) is what we were referring to, and this could reasonably be described as second-order compared to the primary effect of defences on holding back water for events smaller than their standard of protection. However, we appreciate that the wording is clumsy and have therefore deleted the sentence to avoid any confusion.

- Extended data tables and Supplementary data: The results are presented with a lot of significant digits, giving a false sense of their actual precision. I suggest to round these results or add a note about precision and accuracy.

Thanks for the suggestion - we have reduced all tabulated results to 2 significant figures.

Reviewer #2 (Remarks to the Author):

The main objective of this paper is to develop and examine a well-founded set of potential coastal flood exposure scenarios for the UK over the extended period until 2300. The study builds upon two previously developed and published frameworks: one for regional sea-level rise (SLR) storyline construction (Palmer et al. 2024), and another for nationwide estimation of flood-related risks using a high-resolution, hydrodynamically based approach (Bates et al. 2023). The novelty of this work lies in the generation of high-resolution large-scale projections of areas at risk of flooding for time horizons extending beyond the widely accepted and commonly analyzed next century, including, among others, low-probability but high-impact scenarios associated with future ice-sheet instability.

The paper raises and substantiates important questions about the prolonged nature of SLR, the increasing uncertainties in the more distant future due to lacking understanding of underlying physical processes, and the potential implications and shortcomings of short-term planning on the example of the present-day infrastructure and exposure for the UK. Additionally, a notable aspect of physical limitations to the defense facility upgrades under very extreme SLR scenarios was emphasized. The paper is clearly structured, includes relevant references and employs a technical methodology that has been established and validated in previous publications.

Here are some specific comments:
1. While the flood-affected areas and population for the 2300 comprise new information and cannot be cross-validated, it would be useful to have some brief comparison, discussion and references on the previous studies about the change in population exposure due to SLR at least for 2100, where enough studies are available (e.g. Vousdoukas et al. 2020, Nature Comm.).

Thanks for this suggestion. We have made a comparison with the work of Vousdoukas et al (2020), as suggested. Despite the work of Sayers et al (2022, and references therein) clearly being very relevant, we could not find a suitable basis for meaningful comparison.

We have included the following text in the Discussion section and give further details below:

"Our estimated changes in UK population exposure are in broad agreement with previous studies under similar climate scenarios and flood defence assumptions with an increase of approximately 0.5 million for storyline B and approximately 1 million for storyline C by 2100 (Vousdoukas et al, 2020)."

The table below gives a comparison for similar climate scenarios and assumption of no future adaptation measures from Vousdoukas et al (2020) - see their Table 2. Note that the Vousdoukas et al (2020) numbers are based on a 1-in-1-year return level event rather than the 1-in-200-year return level event used in this study, but we expect the increases in population exposure due to sea-level rise to be broadly comparable.

	**Baseline/Present Day**	**RCP4.5 / Storyline B at 2100**	**RCP8.5 / Storyline C at 2100**
Vousdoukas et al (2020) 1-in-1-year event	27,700	461,300<br>(433,600 increase)	1,126,100<br>(1,098,400 increase)
This Study 1-in-200-year event	2,404,812	2,930,051<br>(525,239 increase)	3,396,425<br>(991,613 increase)

2. The absence of defense facilities in the proposed model is discussed and justified. Thus, the results for the affected population provide an upper and improbable limit of possible flood impact. However, it is not mentioned in the abstract, and the numbers could be misleadingly interpreted as risks under the current state of protection.

We agree with the reviewer that this is not clear in the abstract. We have therefore amended the abstract to include the word 'undefended' to make this unambiguous.

3. The study is focused on the impact of SLR. This is not the only, although probably the most substantial, reason for changes in flood exposure. However, for the sake of completeness, it would be helpful to mention potential changes in e.g. storm surge extremes and whether they could be neglected in this context. Another unconsidered aspect is the change in the future population rate and spatial distribution (L. 213), which could alter the results significantly. It is clearly not the purpose of this study, but the

absence of population scenarios could be mentioned and justified either in Introduction or Discussion section.

Regarding the point on storm surges / drivers of coastal floods, we have introduced the following additional sentence to the third paragraph of Main text:

“While coastal flood hazard is also subject to changes in storm surges, waves and tides these are secondary factors compared to the century timescale threat from sea-level rise (Howard et al, 2019) and therefore neglected from this study. ”

The point regarding a static present day baseline for population is a good one and was also raised by reviewer #1 (see response to their major point 3). In response we have added the following text the closing paragraphs of the introductory text (before the Results section):

“We characterise the coastal flood impacts at 2100, 2200 and 2300 associated with these storylines given present day population for the UK as a whole and for the UK capital cities of Belfast, Cardiff, London and Edinburgh”

And

“We work with the present day population as a baseline because currently available UK future projections (Merkle et al, 2022) are: (i) ‘blind’ to flood risk and therefore allow expansion of population into areas which, in reality, would never be developed; (ii) only available at 1km resolution which is insufficiently granular for a localised problem such as flooding; and (iii) only exist out to 2100 as beyond this uncertainties are simply too great. It should be noted, however, that population is not static and future work should incorporate these effects once appropriate projections become available.”

And some questions to the Methodology section:
L 297: “storyline is selected to optimize the fit against individual contributions from…” – not quite clear what was done here. The Monte Carlo realization, most consistent with the selected GMSL percentile for different storylines, was selected. Were there also Monte Carlo simulations for each component (ice sheet, thermosteric contribution, etc.) or which contributions are meant here? Please, either reformulate or provide relevant references.

Thanks. We have revised this section accordingly, including, e.g., the following new text:

“Each Monte Carlo set includes 450,000 simulations of the following contributions to GMSL rise: (i) global thermosteric sea-level rise; (ii) mass addition from Antarctica; (iii) mass addition from Greenland; (iv) mass addition from mountain glaciers; (v) changes in land water storage. Four Monte Carlo sets are used in total The storylines are generated following a two-step process that is designed to provide close consistency with several

literature-based estimates of GMSL rise and the underlying process-based contributions. First, the Monte Carlo set is filtered to retain only those members that match the target GMSL rise at the given time-horizon. Second, a single Monte Carlo member is selected that provides the best fit to the individual contributions (i)-(v).”

L 302: The regression coefficients (global thermosteric to local sterodynamic) were adopted from UKCP18. As far as I know, only standard RCP/SSP scenarios were considered there. What are the coefficients for the H1, H2 storylines and where do they originate from?

Thanks. We have revised the text to be explicit about which regression coefficients were used for which Storylines:

“These values are: 1.372 for SSP1-2.6/RCP2.6 (used for Storyline A); 1.390 for SSP2-4.5/RCP4.5 (used for Storyline B) and 1.465 for SSP5-8.5/RCP8.5 (used for Storyline C, H1, H2).”

L 321: “coastal boundary conditions taken from an Environment Agency extreme value frequency analysis” – are these data used for model validation/calibration? Because it looks like for the Storylines the coastal boundary conditions should be taken from scenarios directly (SLR+extremes).

Thanks for flagging the lack of clarity here. We have revised the text as follows:

“Coastal boundary conditions for the model combine the storyline-based sea-level rise projections with the Environment Agency coastal flood boundary conditions dataset (https://www.gov.uk/flood-and-coastal-erosion-risk-management-research-reports/coastal-flood-boundary-conditions-for-the-mainland-uk-coasts-and-islands) which define the probability-magnitude of meteorologically-driven coastal extreme water levels above mean sea level around the UK.”

L 330-342: The procedure is not entirely clear. Does “hydrodynamics” here refer to the inundation model or to the offshore model to generate coastal boundary conditions? What is meant by “event” – is it a typical surge event with a maximum surge level corresponding to 1/200-year return value? Alternatively, does it refer to the 1/200-year return value from the frequency analysis mentioned in L321-L322, which is linearly superimposed on the SLR Storylines? At what stage is the tidal component included - within the offshore hydrodynamic simulation, if there is any (to capture SLR-tide-surge interactions) or linearly added to (SLR + 1/200-yr extreme) value? Does the shape of the surge curve (duration) play any role for the inundation model, or are the duration and volume of water treated as instantaneous and unlimited?
Clarifying the terms “event” and “hydrodynamic simulation” would likely make the methodology easier to follow.

We agree with the reviewer - although we refer readers to the Bates et al. paper that describes these boundary conditions in more detail, we should have included further information within the manuscript. To answer the reviewer's questions - by hydrodynamics we refer to the inundation model.

By event, we mean that we take a typical surge event with a maximum surge level corresponding to the 1-in-200-year return value of extreme water level (now added explicitly to the same sentence), which includes the impact of tides and wave setup, and then uplift with the addition of sea-level rise as defined by the storylines.

We have amended this in the manuscript to be clearer; with the relevant sentence in the Online Methods section now reading as:

"For this study we re-simulated the inundation hydrodynamics of a 1-in-200-year extreme events for UK coastal regions with peak water elevations increased to represent the impact of the five sea-level rise storylines 2100, 2200 and 2300"

SLR-tide-surge interactions are not represented in this study. This is now explicitly acknowledged in paragraph 3 of the Main text:

"While coastal flood hazard is also subject to changes in storm surges, waves and tides these are secondary factors compared to the century timescale threat from sea-level rise (Howard et al, 2019) and therefore neglected from this study."

The shape of the surge is informed by both the storm surge profiles provided by the Environment Agency and historic tide gauge data; it is not of an unlimited duration.

We have now added the following additional explanation in the manuscript, before directing readers to Bates et al. for further details as per before:

"These extreme water heights were combined with the relevant storm surge profile for the stretch of coastline (as defined by the Environment Agency Design Surge Profile dataset), a time-varying profile based on historic tide gauge data and an estimate of wave setup (calculated using ERA5 reanalysis data) to produce an extreme water height time series for the storm surge event that was simulated by the inundation model."

L 334: seems to be too specific and superfluous for the readers outside the UK

Agreed. We have deleted some of the specifics of this statement and removed reference to the Environment Agency and Natural Resources Wales Flood Zone 3 maps.

The revised sentence now reads as follows:

“We simulated events with a return period of 1-in-200 years as this is the current standard of protection for coastal flood defences in the UK and typically presented in UK national flood risk maps

Reviewer #3 (Remarks to the Author):

In this study, the authors apply a coastal inundation model to compute the present and future area and population exposure to the 1-in-200 year flood event in the UK under different storylines of future sea-level change. I found the presented results interesting and the manuscript succinct and well written. The flood exposure changes that the authors project clearly warrant intensified mitigation and adaptation action.

Having said that, most of the rather general conclusions drawn by the authors appear to be based on the sea-level storylines themselves instead of on the inundation modeling. Furthermore, the novelty of the study is, in essence, a relatively straightforward translation of existing sea-level rise scenarios to exposure changes using an existing inundation model and several simplifying assumptions. Multiple opportunities for scientific advances regarding the exposure modeling were not capitalized on. I therefore think that the manuscript could be revised to increase its currently somewhat limited contribution to the existing literature. I hope my comments below will be helpful to do so.

Whilst this is a very useful challenge regarding our conclusions (and we have made substantive changes to address the reviewer’s comments) we feel it necessary to push back a little on this comment. Using the storylines alone would mean that it would be impossible to produce the inundation maps in Figures 2 and 3 and the population exposures in Figure 4. Neither would it be possible to have the detailed discussion on impacted major UK infrastructure that constitutes the bulk of the results section (e.g. the whole block of text from lines 170-242 in the original manuscript). Further, it is normal and expected that the Discussion section seeks to generalize these detailed findings to larger scale policy implications.

We would also make the point that going from storylines to exposure is only a ‘relatively straightforward translation’ if one has spent multiple person-years developing a high-resolution national-scale flood inundation model that can do this. This is reported in Bates et al (2023) and represents, to the best of our knowledge, the first ever peer-reviewed catastrophe risk model for flood anywhere in the world. Even then, implementing the extreme boundary conditions considered here is beyond normal catastrophe risk modelling, and re-running national scale simulations requires significant deployment of high-performance computing effort. We do not believe that this is straightforward in any way and is only possible for very few research teams globally.

Main comments

1. I find a large part of the Discussion (and conclusions) section too general. In my opinion, most of the key conclusions in L229-270 could have already been drawn without the

inundation and exposure analysis. For instance, the conclusion that “this combination of sea level rise allowances and residual uncertainty allowances should therefore likely accommodate the values of sea-level rise for the UK across all storylines that have peak values of about 1.5 m” (L237-239) is based purely on the sea-level storylines and system knowledge, not on the exposure changes. The same argument applies to most of L254-270. While the points that the authors raise here are clearly valid and relevant, they do not relate to the results of this study directly enough and overlap with the main messages of previous studies as well (e.g., Palmer et al., 2024; Turner et al., 2025, Slangen et al., 2022, etc.). I would therefore encourage the authors to provide a more detailed discussion of their inundation/exposure modeling results, clarify what specifically these results mean for mitigation and adaptation planning in the UK, and include a discussion of how their results compare to previous flood exposure studies, which is currently lacking.

Thanks for highlighting this short-coming in the original text. We have revised this section substantially in-line with the reviewer’s suggestions.

In paragraph 1 of the discussion, we have added the additional text (see also response to reviewer 2 on population exposure comparisons):

“Under Storyline B, the 2100 population exposed to flooding increases by 25%, which is slightly below the 27% increase in properties at risk by 2069 predicted by the most recent UK National Flood Risk Assessment (NaFRA2). Therefore, our exposure storylines are broadly consistent while being conservative with respect to UK government projections this century. The estimated changes in UK population exposure are similar to previous studies under similar climate scenarios and flood defence assumptions with an increase of approximately 0.5 million for Storyline B and approximately 1 million for Storyline C by 2100 (Vousdoukas et al, 2020).”

We also refer to post-2100 population exposure changes in paragraph 2 of the Discussion, focusing on the range of outcomes across storyline A and storyline H2, as follows:

“Storyline A clearly illustrates the huge potential for avoided sea-level rise through swift and substantive global greenhouse gas emissions reductions consistent with Paris Agreement targets, with exposure increasing by only 28%. Conversely, storyline H2 illustrates the vast threat of sea-level rise for the UK if ice sheet instability processes are triggered with values that far exceed the current UK H++ credible maximum scenario of 1.9 m within a few decades of 2100 and a five-fold increase in population exposure by 2300 - vastly outside the range reported in NaFRA2.”

The revised paragraph 3 of the Discussion addresses the reviewer’s comments on mitigation and adaptation planning:

“Our results show many instances of major UK infrastructure moving into flood risk areas as sea level continues to rise beyond 2100. Increased exposure to coastal flooding is identified across all storylines. Under storyline B, approximately half of England’s coastal

flood defences would need investment over the subsequent 200 years to maintain defence standards, with a small number potentially being beyond hard adaptation limits (Table S6). Under storyline H2, >99% of flood defences could be beyond hard adaptation limits by 2200 (Table S6). The increased population exposure to the 1-in-200-year flood event under the H2 storyline increases from ~3 million to almost 10 million between 2100 and 2200 implies widespread disruption to the economy and society, with challenges compounded by potentially reaching hard adaptation limits (Table 6). This scenario would require high levels of investment for both new and existing flood defences, moving of communities away from coastlines and put critical infrastructure and sites of national importance at high risk of disruption.

It is therefore inevitable that a combination of additional or enhanced flood defences and managed retreat will be necessary to mitigate against post-2100 flood risk. Stakeholders who only consider changes over the coming century may have a false impression of the true risk landscape given the potential for post-2100 acceleration of sea-level rise. Planning of long-lived infrastructure and future settlements must therefore be cognizant of the full range of possible outcomes for multi-century and longer-term committed sea-level rise to avoid costly future interventions."

2. L17-18, L266-268, L275-276: the authors conclude from their analysis of flood exposure that under storylines with high sea-level rise, 'maintaining current coastlines will no longer be economically viable for most of the UK' and that this 'necessitates large-scale movement of settlements, population and infrastructure'. These conclusions are not directly supported by the analysis. Namely, the authors only compute changes in hazards and exposure to the 1-in-200 year event, not in overall impact and risk. Furthermore, they do not provide any cost/benefit analysis of different adaptation strategies (as in, e.g., Tiggeloven et al., 2020, Ballesteros et al., 2025). Finally, as seen in countries already strongly impacted by sea-level rise, the lack of economic viability of coastal protection does not necessarily mean that migration and retreat is needed or will follow (e.g., Esteban et al., 2019). I think these aspects need to be incorporated.

We agree with the reviewer that this research has focussed on exposure and hazard and does not provide a full cost-benefit analysis, which is clearly beyond the scope of the present study. However, we have expanded on this point as part of the discussion and toned-down the text in light of the reviewer comments, stating:

"For multiple metres of sea-level rise, maintaining current coastlines would likely prove extremely challenging for most of the UK and would therefore likely necessitate large-scale movement of settlements, population and infrastructure away from the coast"

We note that Reviewer 1 endorses this important message from our findings in their own review.

We have added additional analysis that demonstrates how nearly all Environment Agency defence heights will be overtopped by > 2 m by 2200 for the H2 scenario, indicating that significant investment would be required to mitigate against this risk, justifying the text above.

We have also added a call for a national scale cost benefit analysis at the end of this paragraph, referencing the studies mentioned:

"*Due to the country-wide connectivity of vital infrastructure, it is imperative that full cost-benefit analyses (Tiggeloven et al., 2020, Ballesteros et al., 2025) are undertaken at the national scale to fully understand the economic, political and societal impacts of coastal flooding under such extreme future sea-level rise scenarios.*"

We have also made a minor text change in the abstract to reflect that the movement of populations away from the coast is a possibility, rather than a certainty.

"This would imply the potential need for large-scale movement of populations and settlements away from the coast in the coming centuries."

References:
Tiggeloven, T., de Moel, H., Winsemius, H. C., Eilander, D., Erkens, G., Gebremedhin, E., Diaz Loaiza, A., Kuzma, S., Luo, T., Iceland, C., Bouwman, A., van Huijstee, J., Ligtvoet, W., and Ward, P. J.: Global-scale benefit–cost analysis of coastal flood adaptation to different flood risk drivers using structural measures, Nat. Hazards Earth Syst. Sci., 20, 1025–1044, https://doi.org/10.5194/nhess-20-1025-2020, 2020.

Ballesteros C, Lincke D, Nicholls RJ, Heslop J, Hinkel J, Malagón-Santos V and Slangen ABA (2025) Migration, land loss and costs to 2100 due to coastal flooding under the IPCC AR6 sea-level rise scenarios and plausible adaptation choices. Front. Mar. Sci. 12:1505633. doi: 10.3389/fmars.2025.1505633

3. The authors make a lot of simplifications in their modeling approach that, in my view, detract from the scientific value of their study:
a. L302-304: existing analyses with both global and regional climate models suggest that (1) the pattern of sterodynamic sea-level change around the UK is not spatially uniform (e.g., Fox-Kemper et al., 2019; Hermans et al., 2020) and that (2) the relationship between global mean thermal expansion and regional to local ocean dynamic sea-level change is not necessarily linear, especially not on the longer timescales considered in this manuscript (Malagon-Santos et al., 2023, 2025). It would help to address these aspects or at least justify their neglection.

Thanks for the comment. We have taken the approach to remain consistent with the basis of UK sea-level rise information as a priority for this study, i.e., the methods used in UKCP18. Introducing methodological differences would detract from the immediate

relevance of our study to UK sea-level rise planning processes by breaking the connection to the underlying projection framework.

On the reviewer's first point, we would argue that the work of Hermans et al (2020) supports the use of a spatially uniform pattern of sterodynamic sea-level change around the UK - the downscaled simulations that better resolve bathymetry and include representation of tides are much more spatially uniform than the "parent" global climate model simulations.

On the reviewer's second point, Malagón-Santos et al (2023) show that the approach to characterising the relationship between global thermal expansion and local sterodynamic sea-level rise yields differences on the order of centimeters. This is two orders of magnitude smaller than the multi-century global and local sea-level changes considered here and clearly would not substantively alter the results presented.

Both of the reviewer's points are addressed in the revised Supplementary Materials, including plots of the relationship between global mean thermal expansion and local sterodynamic sea-level change. For convenience, we include the relevant text below:

*"**Note S2: Approach to local sterodynamic sea-level rise***

Following the methods presented in UKCP18 (Palmer et al, 2018), a scenario-specific space-and-time invariant regression coefficient is used to estimate the local sterodynamic sea change for each storyline, as described in the main text. The assumption of spatial invariance was originally made in UKCP18 because the underlying CMIP5 global climate model (GCM) simulations have limited horizontal and vertical resolution and are missing key processes, such as tides (e.g., Tinker et al, 2020). Therefore it was concluded that any spatial variations around the UK coast were unlikely to be robust and that an assumption of spatial uniformity was more appropriate (Palmer et al, 2018). Subsequently, Hermans et al (2020) have shown that downscaling of CMIP5 GCM simulations results in essentially spatially uniform patterns of sterodynamic sea-level change round the UK coastline.

Under UKCP18, scenario-specific regression coefficients for each RCP scenario were computed on the basis of a linear fit between global thermosteric sea-level rise and local sterodynamic sea-level rise for CMIP5 model simulations over the period 2007-2100 (Figure S1). The same process was carried out for each CMIP5 GCM grid box adjacent to the UK coastline. Given the assumption of spatial invariance noted above, a central estimate value was used across all available CMIP5 UK coastal grid boxes. It was assumed that these regression coefficients were representative of the time-horizon out to 2300, which is a reasonable first-order approximation in most cases, as illustrated for the available CMIP5 simulations under the RCP4.5 scenario (Figure S2). Further details are available in Palmer et al (2018)."

b. The authors ignore socioeconomic developments which, especially at multi-centennial timescales, can also be an important driver of changes in exposure (e.g., Hinkel et al., 2021; Lincke et al., 2022; Ballesteros et al., 2025). It could be insightful to include scenarios of, for instance, population changes in the exposure modeling, or at least their

omission should be more elaborately discussed in the context of similar studies that do include such changes.

We agree that this is a very important topic but, as we have noted previously, the only spatially detailed UK population and socio-economic projection to 2100 has significant limitations for this type of analysis (it is 'blind' to flood risk, insufficiently granular and does consider storylines H1 and H2). No credible projections at all exist for the period after 2100.

As a result, we don't believe it is yet possible to do the analysis the reviewer suggests, although it would be incredibly interesting. We have now added a more thorough discussion of why we neglect this aspect in the introduction:

"We characterise the coastal flood impacts at 2100, 2200 and 2300 associated with these storylines given present day population for the UK as a whole and for the UK capital cities of Belfast, Cardiff, London and Edinburgh"

And

"We work with the present day population as a baseline because currently available UK future projections (Merkle et al, 2022) are: (i) 'blind' to flood risk and therefore allow expansion of population into areas which, in reality, would never be developed; (ii) only available at 1km resolution which is insufficiently granular for a localised problem such as flooding; and (iii) only exist out to 2100 as beyond this uncertainties are simply too great. It should be noted, however, that population is not static and future work should incorporate these effects once appropriate projections become available. "

Reference:
Merkle, M. et al. Downscaling population and urban land use for socio-economic scenarios in the UK. Reg Environ Change 22, 106 (2022).

c. It is unclear if/how the authors account for the uncertainty in the height of the 1-in-200 year return event, which could be comparable to the uncertainty of mean sea-level rise (Wahl et al., 2017). Including this uncertainty could be a relatively straightforward step to increase the novelty of the study.

Thanks for this suggestion. The uncertainty in the height of the 1-in-200-year return event is not represented. We decided to take the central estimate from the Environment Agency in determining the height of the 1-in-200 year event as we were interested in assessing the relative impacts as a result of SLR and were limited with the computational resource available for simulations. We have made this point explicit in the Online Methods section:

"For this study we re-simulated the inundation hydrodynamics of a 1-in-200- year extreme event (based on the central estimate) for UK coastal regions with peak water elevations

increased to represent the impact of the five sea-level rise storylines 2100, 2200 and 2300."

The main point of our study is to explore how future sea-level rise could exacerbate a given extreme flood event and the storyline framework we have deployed does not explicitly include uncertainty information.

In any case, indicative values for the uncertainties for the 1-in-200-year return level around the UK is about +/- 10-20 cm. Much smaller than the range of sea-level rise outcomes explored here. However, we agree that this could be a useful topic for future studies, either through exploring the full uncertainty space, or considering change across events of different magnitudes.

A related point are dynamic changes in tides, waves and surges, which could substantially affect the changes in the exposure as well. How valid is it to ignore these changes on these long timescales? And could it be useful to incorporate storylines of such changes as well based on previous work by the authors (e.g., https://iopscience.iop.org/article/10.1088/2515-7620/ad3368)?

Yes - this is a good point that was also raised by other reviewers. In response to the first aspect we have added the following in the third paragraph of the Main text:
"While coastal flood hazard is also subject to changes in storm surges, waves and tides these are secondary factors compared to the century timescale threat from sea-level rise (Howard et al, 2019) and therefore neglected from this study. "

In regards to the reviewer's second point, yes - one could add other elements to our storyline framework (such as the potential for AMOC weakening leading to increased storminess). This is beyond the scope of the current work but is in the scope of work currently planned by our wider research teams.

d. Adaptation measures (present or future) were not incorporated. The authors' motivation that this is a 'second-order factor' (L338) is clearly incorrect: in fact, it is one of the most important factors in assessments of coastal flood risk, if not *THE* most important factor (Hinkel et al., 2021). I understand that including current and future adaptation measures involves substantial additional uncertainties, but assessing different adaptation scenarios does provide useful insights (e.g., Tiggeloven et al., 2020; Ballesteros et al., 2025) and in my view, the authors need to discuss this caveat of their work more prominently in the main text.

This point was also raised by Reviewer #1 and apologies that our phrasing here was unclear. We did not intend that flood defences should be considered a second order factor. Rather, what we meant was that even an overtopped flood defence can still have a minor continuing impact on flood extent compared to the situation where there is no defence present at all. This effect (impact of over-whelmed flood defences on flood propagation) is what we were referring to, and this could reasonably be described as

second-order compared to the primary effect of defences on holding back water for events smaller than their standard of protection. However, we appreciate that the wording is clumsy and we have deleted the sentence to avoid any confusion.

We fully agree that adaptation is critical to the impacts of future sea-level rise but, as we note elsewhere in this response, there are no credible UK socio-economic projections out to 2300 that would support this analysis at present. We now pick this point up in the Discussion where we call for the development of such scenarios that could allow detailed analysis of adaptation pathways in the face of such extreme future sea-level rise. This is critical as it is clear that current national decision making frameworks, centred on the period out to 2100, are likely to be inadequate for post-2100 time-horizons.

We have added the following text as part of the Discussion:

"Due the the country-wide connectivity of vital infrastructure, it is imperative that full cost-benefit analyses (Tiggeloven et al, 2020; Ballesteros et al, 2025) are undertaken at the national scale to fully understand the economic, political and societal impacts of coastal flooding under such extreme future sea-level rise scenarios. This type of assessment would require a set of plausible scenarios that include feedbacks between population dynamics and the evolving flood hazard under adaptation measures and future sea-level rise that extend into the coming centuries."

4. Apologies if I missed it, but I cannot find any code or data availability statement. This would render the study irreproducible and does not comply with FAIR principles.

Apologies, this was an oversight at the time of submission. We have included a Data Availability section as part of the revised manuscript that reads as follows:

"Data Availability

The data that support the findings of this study are available at https://doi.org/10.5281/zenodo.18862415. This archive also includes the essential third-party datasets: (i) Environment Agency extreme water level dataset; (ii) the Digital Elevation Model; and (iii) the WorldPop population dataset. The sea-level rise storylines used in this study are available at https://zenodo.org/records/18419404.

The coastal boundary condition dataset can be downloaded from the Environment Agency website directly at https://environment.data.gov.uk/dataset/84a5c7c0-d465-11e4-b0bd-f0def148f590. Supporting documentation on how to use this data can be found on the UK government website: https://www.gov.uk/flood-and-coastal-erosion-risk-management-research-reports/coastal-flood-boundary-conditions-for-the-mainland-uk-coasts-and-islands. LiDAR data can be found on the UK Government websites at the following locations: England https://environment.data.gov.uk/dataset/09ea3b37-df3a-4e8b-ac69-fb0842227b04
Scotland https://remotesensingdata.gov.scot/data#/list

Wales https://datamap.gov.wales/maps/lidar-data-download/
Northern Ireland
https://admin.opendatani.gov.uk/dataset/?tags=Elevation&tags=Digital+Terrain+Model&_tags_limit=0

OS Open Terrain can be downloaded at
https://osdatahub.os.uk/data/downloads/open/Terrain50
Tide gauge data for the UK can be found on the BoDC website
https://www.bodc.ac.uk/data/
ERA-5 reanalysis data can be downloaded on the Copernicus website here:
https://cds.climate.copernicus.eu/datasets/reanalysis-era5-single-levels
WorldPOP data is available at https://www.worldpop.org/
Merit-Hydro is available for academic purposes at https://hydro.iis.u-tokyo.ac.jp/~yamadai/MERIT_Hydro/

Code Availability

The code used to generate Figures 1 and 4 (and associated supplementary figures) is publicly available at https://doi.org/10.5281/zenodo.18862415.

Figures 2 and 3 were produced in QGIS using version 3.22.4. The QGIS software is freely available from https://qgis.org/

The LISFLOOD-FP code used for the flood inundation modelling is open source and available at https://zenodo.org/records/13121102."

Other Comments
General: None of the URLs referred to in the paper worked for me, even though some of these references contain information key to the study. I would recommend the authors to appropriately cite these references, including access dates, and to make sure this information will remain accessible in the future.

Apologies that the URL links didn't work. This was due to how the software was failing to text-wrap, which caused problems when the conversion to PDF was made. We have replaced the hyperlinks in the text with citations, as requested. We will include all essential third-party datasets in the Zenodo repository described in the Data Availability Statement (see above).

Figure 1: Different scales are used for each map, which is somewhat confusing since the same colormaps are used. Please consider if a single logarithmic scale could be helpful.

We have revised Figure 1 and Extended Data Figures 1 and 2 (now Supplementary Figures 1 and 2) so that different colormaps are used for each unique colorscale. We opted to retain the original linear colorscales (with minor modifications in some cases) to preserve the spatial patterns in each sub-panel. We could not find a solution for a single logarithmic scale that preserved the underlying information.

L112-L159: A substantial part of the Results section introduces results of a previous study by the authors (Palmer et al., 2024, ref. 9), not results of the present study, which I found misleading. While I understand this is important background information, the same information is also presented in the Online Methods. Perhaps the paragraphs in the main text could therefore be condensed to a few sentences, and a table or subfigure that briefly summarizes the characteristics of each storyline could be added to Figure 1?

We have given thorough consideration to the reviewer's comments. We are hesitant to remove the contextual information presented that is vital for the reader to fully understand the flood modelling results that follow. In particular, understanding the narrative elements of the storylines is essential to the interpretation of the adaptation vs mitigation aspects of this study (and what aspects of future flood hazard are potentially avoidable).

These narrative aspects are NOT included on the Online Methods, which focus on the technical aspects of how the numerical values of sea-level rise were created for each storyline. So we do not agree that the "same information is presented in the Online Methods".

Figure 1 is designed to be a concise synthesis of the SLR storylines and characterizes both their temporal evolution (Panel a) and spatial variations across the UK domain (Panels b-f). We prefer to keep this figure as it is.

As a compromise, we have moved the narrative elements of the sea-level rise storylines that were in the main text into a new Table 1. Hopefully this satisfies the reviewer's request to condense the information and we think this is probably a more helpful layout for the reader to compare across the various storylines and we thank the reviewer for this suggestion.

L133-134: The bracket notation is slightly confusing – please consider spelling this out for clarity.

Thanks. We have revised the sentence to remove the bracket notation.

L166-167: Since the authors do not consider flood defenses in their analysis, it is unclear to the reader what this statement is based on. Would it be helpful to add a few examples?

Thanks for your comment. In hindsight, the text here wasn't very clear and potentially confusing. We have simplified the text to read as follows:

"Given climate change and uncertainty tolerances within flood defence design (https://www.gov.uk/guidance/flood-risk-assessments-climate-change-allowances#sea-level-allowances; https://www.gov.uk/flood-and-coastal-erosion-risk-management-research-reports/accounting-for-residual-uncertainty-an-update-to-the-fluvial-freeboard-guide), it is likely that the real-world impacts of a ~0.5 m sea-level rise in areas protected by coastal flood defences would be limited."

Separately we have further explored the likely impact on flood defences in the new supplementary section S3 and Table S6. These results are discussed in the final paragraph of the Results section in the revised manuscript.

Matt to address.
L297: "optimize the fit against" I didn't understand what this means, sorry. Could you please clarify?

MP: The text has been revised to clarify the description of the Monte Carlo simulations. We include the revised text below - the last sentence is the one that directly addresses the reviewer comment.

"Each Monte Carlo set includes 450,000 simulations of the following contributions to GMSL rise: (i) global thermosteric sea-level rise; (ii) mass addition from Antarctica; (iii) mass addition from Greenland; (iv) mass addition from mountain glaciers; (v) changes in land water storage. Four Monte Carlo sets are used in total The storylines are generated following a two-step process that is designed to provide close consistency with several literature-based estimates of GMSL rise and the underlying process-based contributions. First, the Monte Carlo set is filtered to retain only those members that match the target GMSL rise at the given time-horizon. Second, a single Monte Carlo member is selected that provides the best fit to the individual contributions (i)-(v)."

L321-L334: these lines discuss the extreme sea-level analysis used as input to the inundation model. Besides that the URL referred to does not seem to work, this is methodological information that in my opinion should be included in the article itself, especially given the central role of the 1-in-200 year return event in this study.

Thanks for this comment - we agree that return level information is crucial to the study. We have clarified this in the second-last paragraph of the Main text, which reads as follows:

"Simulations are based on the 1-in-200-year return period extreme coastal water level event from the UK Environment Agency coastal flood boundary condition dataset (see Online Methods) since this is the typical design standard of UK coastal flood defences."

The Online Methods section includes the following additional information:

"Coastal boundary conditions for the model combine the storyline-based sea-level rise projections with the Environment Agency coastal flood boundary conditions dataset

(https://www.gov.uk/flood-and-coastal-erosion-risk-management-research-reports/coastal-flood-boundary-conditions-for-the-mainland-uk-coasts-and-islands) which define the probability-magnitude of meteorologically-driven coastal extreme water levels above mean sea level around the UK. These extreme water heights were combined with the relevant storm surge profile for the stretch of coastline (as defined by the Environment Agency Design Surge Profile dataset), a time-varying profile based on historic tide gauge data and an estimate of wave setup (calculated using ERA5 reanalysis data) to produce an extreme water height time series for the storm surge event that was simulated by the inundation model. Further details of the boundary condition and model discretisation can be found in Bates et al[20] who showed that this model could skilfully predict present day flood areas and water depths when compared to UK Government official flood maps and post-event water depth observations."

L341-342: It was not clear to me what this refers to, does this mean that surges and tides are linearly combined and if so, how valid is that assumption?

Yes - the reviewer is correct: we assume that surges and tides combine linearly. We have added the following text in this section to acknowledge this assumption (which is supported by past literature):

"Our approach is supported by previous studies that have shown that mean sea level, tides and surge combine linearly, to a good approximation (Howard et al, 2010; Palmer et al, 2018)."

References other than already cited:

Ballesteros et al. (2025) https://doi.org/10.3389/fmars.2025.1505633
Esteban et al. (2019) https://doi.org/10.1016/j.ocecoaman.2018.10.031
Hermans et al. (2020) https://doi.org/10.1007/s00382-019-05104-5
Howard et al. (2010) https://doi.org/10.1175/2010JCLI3520.1
Hinkel et al. (2021) https://doi.org/10.1029/2020EF001882
Lincke et al. (2022) https://doi.org/10.1029/2021EF002584
Tiggeloven et al. (2020) https://doi.org/10.5194/nhess-20-1025-2020
Turner et al. (2023) https://doi.org/10.1029/2023EF003550
Malagon-Santos et al. (2023) https://doi.org/10.5194/os-19-499-2023
Malagon-Santos et al. (2025) https://doi.org/10.1088/1748-9326/ade906
Slangen et al. (2022) https://doi.org/10.1029/2021EF002576
Wahl et al. (2017) https://doi.org/10.1038/ncomms16075

We would like to thank all the reviewers for their constructive comments on our manuscript. Reviewer 4 was particularly helpful in challenging us to further consider the framing and implications of our study.

Throughout this document, our responses to reviewer comments appear in **blue** and the reviewer comments appear in **black**.

REVIEWER COMMENTS

Reviewer #1 (Remarks to the Author):

I thank the authors for responding satisfactorilly to my previous comments. I only have one final minor comment: given the modelling framework used by the Authors to evaluate total water levels at the coast, I am not sure that waves are taken into account. This means that in the final paragraph of the discussion, the defenses would not be overtopped but rather overflown by approximately 1m, which indeed would generate a major flood event. This is because many coastal engineers define overtopping as mean total water levels being below flood defenses, but waves generating flooding beyond coastal defenses. I just recommend to check this terminology point and adjust.

I hope it is useful

Gonéri le Cozannet, BRGM, 16th March 2026

Thank you for the very useful comment - this has now been reworded to remove the ambiguity around overtopping and overflow. The sentences now reads:

"Under Storyline B, in 2100, < 1% of defences are modelled to have a maximum water level exceeding the defence height by 1 m or more. This increases to 27% by 2200 and 35% by 2300. Comparatively under Storyline H2, in 2100, 10 % of defences are modelled to have a maximum water level exceeding the defence height by 1 m or more, increasing to nearly 100 % by 2200"

Reviewer #2 (Remarks to the Author):

The main questions are clarified satisfactorily.

The second draft benefits from an additional consideration of coastal defense crest heights and corresponding statistics on overtopping. This illustrates SLR potential impacts, especially for high-end storylines, in a more comprehensive and relatable way.

Minor comment:

Supplement Note S2 and related reply to Rev.1: it looks like here the explanation about the space-time invariant coefficients for sterodynamic regional sea-level rise is relevant only for the thermosteric part of GMSL rise (i) and not for the entire GMSL/RMSL relation, as also described in the main manuscript. Otherwise, it would contradict the regional spatial variability presented in Fig.1 and following discussion. So, in the present form the title of the Note2 is misleading and should somehow include “thermosteric GMSL” term to underline only partial space-time uniformity.

Apologies that our explanation has caused some confusion. We have revised the text to make it more comprehensive and explicit in the steps taken (see changes below). The reviewer is correct: the space-time invariant coefficients only apply for the conversion of global thermosteric sea-level rise to sterodynamic sea-level change.

The spatial patterns seen in Figure 1 and discussed in the manuscript arise from (i) the GRD patterns associated with changes in glaciers, ice sheets and land water storage; and (ii) the effect of GIA on local relative sea-level change.

In the main text Methods section we have clarified the text in two aspects:

(1) We make clearer that the localisation process has several elements by denoting a first, second and final step. We have made it clearer that the first and final steps include spatial patterns.
(2) that the timeseries of global thermosteric sea-level change are converted to a representative UK-average sterodynamic sea-level change that is spatially uniform.

In Supplementary Note S2 we have expanded the explanation and included specific references to the CMIP variables used in the calculations to make clear that this only applies the to the sterodynamic part of sea-level change:

“Following the methods presented in UKCP18, a scenario-specific space-and-time invariant regression coefficient is used to estimate the local sterodynamic sea-level change for each storyline. For each CMIP5 model the linear best fit between local sterodynamic sea-level change (CMIP variables: zos + zostoga) and global thermosteric sea-level change (CMIP variable: zostoga) is computed for every UK-adjacent model grid box (see Palmer et al 2018 for further details). For each scenario, we take an average across all CMIP5 model regression coefficients that ensures each model is weighted equally. This results in a single regression coefficient for each scenario that represents the average for the UK across the CMIP5 ensemble”

Our terminology follows that proposed by Gregory et al (2019) and represents community best practice. They define stereodynamic sea level change as:

“This term is the sum of ocean dynamic sea-level change and global-mean thermosteric sea-level rise”.

Therefore, we find the title of Supplementary Note S2 (Approach to local sterodynamic sea-level rise) to be accurate.

Reviewer #2 (Remarks on code availability):

Could't access the page with the message "Permission required"

> Apologies for this. We were reluctant to make the repository open access at this stage of the review process but passed a direct link to the Editor. We presume that this was not passed on to the reviewers. We will make the repository open access once the paper has been accepted for publication.

You can access the Zenodo repository directly via this link (we have tested this link and understand it to be working):
https://zenodo.org/records/18862415?preview=1&token=eyJhbGciOiJIUzUxMiJ9.eyJpZCI6ImUwOGExNmU5LTE1MDYtNGRkMC04ZmIxLTY3M2JmMWUzMjFjYSIsImRhdGEiOnt9LCJyYW5kb20iOiJlOGU0YzcwMTcwOGQyZWJlMDdmZjY3ZmJmZmM4OTY3YSJ9.Hrd6FzvYSBm1hcxkBJiC_IbC5KoKnqFBSINuPZSHpyzLRGVnwIrufzkxWRbxY3nHklpr13Z5aVJmNLSsxJcCoQ

Reviewer #4 (Remarks to the Author):

This paper extends earlier analyses of the implications of sea-level rise for the UK. It links the sea-level rise outputs from IPCC AR6 to 2300 and also considered the national coastal flood implications to 2300. This compares to earlier analyses which only extended to 2100.

The analysis is credible and interesting in a technical sense. However, with any such analysis there are inevitably -- so what? -- or --what if? -- kinds of questions. Below I pose the key questions that spring to mind. I also identify a few smaller points in the manuscript.

Once these are addressed, I would like to see the authors responses and expect to be happy to see this paper published.

1. What is a meaningful reasonable worse case? The high-end scenarios of IPCC AR6 assume high emissions linked to the RCP8.5 emissions scenario. These are considered rather unlikely and yet are often analysed like they are business-as-usual (e.g. Hausfather and Peters, 2020). So there is an unlikely emissions scenario driving the H1 and H2 storylines. And these outcomes are associated with one model available to the AR6 or one line of evidence. In contrast, the earlier H++ scenario was associated with multiple lines of evidence --- process models, palaeoclimate analogues, semi-empirical models, etc. all seemed to justify a number around 2 m in 2100. Hence, when you come to a reasonable worst case as you define it, which is a “challenging manifestation of the scenario after highly implausible scenarios are excluded” what are the highly implausible scenarios you

have excluded? It might be argued that you have taken the biggest number you have got? So why not consider the H2 as the reasonable worst case? Note that another line of evidence can support it – van de Wal et al (2022) -- which is independent of the IPCC AR6 analysis. At the end of the day, this is down to expert judgement but a little more justification of why you pick H1 over H2 would be good including acknowledging issues around emissions. And is it possible to have two reasonable worst cases – the H1 and H2 cases? So please review the paper in the light of the above points and improve your justification for your selection, including acknowledging RCP8.5 is an unlikely scenario and the high end scenarios are exploratory.

Thank you for this useful perspective. In light of the reviewer's comments we have made the following changes to manuscript:

(i) We have added the following text to the Introduction:

"While we acknowledge that high emissions futures are considered unlikely (Hausfather and Peters, 2020), the sea-level rise outcomes associated with simulations run under these scenarios are important for more complete understanding the total risk landscape and for risk-averse decision contexts (Kopp et al, 2023; Stott et al, 2026)."

(ii) We have revised the text to better justify why we focus on H2 for the flood maps presented in Figures 2 and 3:

"While both H1 and H2 could be used as a reasonable worst case scenario, we focus on H2 because it corresponds to the Low-Likelihood High-Impact storyline that was given prominence in the IPCC AR6 Summary for Policymakers and more completely illustrates the risk that could be avoided through effective global action to reduce greenhouse gas emissions (Stott et al, 2026)."

(iii) We think that the definition described in Supplementary Note S1 remains appropriate for our study, but we now cite Kopp et al (2023) where readers can find more details on the discussion of MICI (and the importance of the H2/LLHI Storyline highlighted in AR6 in decision-making contexts). The text there reads:

"Evaluations are made using both quantitative data (where possible) and expert judgement (where not), with the latter approach more typical for lower probability risks. The likelihood of Marine Ice Cliff Instability (MICI) occurring at scale by 2300, which would create scenarios of extreme sea-level rise, is currently poorly known but cannot be credibly ruled out."

And we have added the following text to the final sentence:

"... as assessed in IPCC AR6 (Fox-Kemper et al, 2021) and further discussed by Kopp et al (2023)"

Additional References:

Kopp, R. E. *et al.* Communicating future sea-level rise uncertainty and ambiguity to assessment users. *Nat. Clim. Change* **13**, 648–660 (2023).
Stott, P. A. *et al.* We need a global assessment of avoidable climate-change risks. *Nature* **650**, 826–828 (2026).

2. What is the purpose of the analysis? It states it is to inform adaptation planning although first and foremost it informs on exposure and risk. In terms of the interpretation of exposure and risk, over the next century or so it will inform adaptation. But beyond this it is informing wider climate policy as we would like to avoid an H1 and H2 world if at all possible. This gives more impetus to climate mitigation although adaptation is an inevitable part of the mix with the UK already having large coastal floodplains. It might also raise thoughts about geoengineering interventions in Antarctica (e.g., Leane and McGee, 2020; Moore et al., 2021). I am not a fan of these proposals, but they are out there and if you raise catastrophic changes these mega interventions will also be raised. So please review the stated purpose of the paper and how it realistically informs policy throughout the manuscript.

Thank you for your helpful challenge on the purpose of the paper. We agree that the mitigation aspect could come across more clearly and have added the following sentence to the end of the manuscript:

"The key policy implications of our study are to inform near-term adaptation planning for the period out to 2100 and further motivate rapid and deep reductions in global greenhouse gas emissions to mitigate against the risk of accelerated sea-level rise from ice sheet instability processes in the centuries that follow."

The mitigation aspect is also touched upon earlier in the manuscript in response to your earlier review comment:

"While both H1 and H2 could be used as a reasonable worst case scenario, we focus on H2 because it corresponds to the Low-Likelihood High-Impact storyline that was given prominence in the IPCC AR6 Summary for Policymakers and more completely illustrates the risk that could be avoided through effective global action to reduce greenhouse gas emissions (Stott et al, 2026)"

While we appreciate the reviewer's thoughts, we consider discussion of geoengineering interventions beyond the scope of this paper. In terms of the policy implications, we have made these more explicit through the additional text quoted above.

Furthermore, we argue that the policy implications are made clear in the sections on "**Impacts of sea-level rise on UK coastal flood hazard**" - where we discuss low-lying areas of the UK and specific national infrastructure assets that could become vulnerable in future, concluding with an analysis of overtopping of current defences - and in the "**DISCUSSION**" - where we we refer to, for example, limitations of current UK planning guidance and discuss the potential need for large-scale movement of settlements, population and infrastructure away from the coast.

3. For the H2 scenario in 2300 why do you not also consider inundation – i.e. those areas that would be below normal high tides so underwater almost everyday without defences. The analysis talks about flooding – but for much of the flooded area, the flood would never go away. So please make sure that the manuscript conveys this important point.

Thanks for raising this important point. We have added the following sentence in the section on "**Impacts of sea-level rise on UK coastal flood hazard**":

"Although we have not attempted to model changes in coastlines, Storyline H2 at 2300 would see large areas of low-lying land below the nominal high tide line and therefore permanently inundated."

4. The Environment Agency only cover England and this should be indicated to the reader when this is used (e.g. Supplemental Table S8).

> Thanks for raising this important point. We have included the following additional text in Supplementary Note S3:

"We note that the Environment Agency AIMS dataset, and hence our analysis, is limited to flood defences in England."

We also make explicit reference to the analysis referring to flood defences in England in the main text (final paragraph of RESULTS) and in the caption for Supplementary Table 8.

Smaller points
L31 – 'primary threat' – there is no other threat of multi-metre SLR except the two big ice sheets so this needs rewording.

> We have removed "multi-metre", so that the sentence simply reads: "… these huge ice reservoirs remain the primary threat for sea-level rise in the coming centuries".

L54-57 – this statement is true but so what? --- it is comparing a 2100 projection to 2300 projections– think about the point and rephrase – big rises are possible as we extend timescales beyond a century?

> Thanks for the helpful challenge here. We have revised the text to read as follows, emphasising the nearer 2150 time-horizon and citing specific results for London from Palmer et al (2024):

"… yet sea-level rise in London could reach 5 m by 2150 and more than 15 m by 2300, based on outcomes highlighted in IPCC AR6[2,8] (Palmer et al, 2024)."

L91-92 1 in 200 standard is the typical design standard for urban areas – less densely populated areas would receive lower protection standards and maybe nothing depending on what is exposed.

Thanks. We have clarified this point so that the text reads:

"since this is the typical design standard of UK coastal flood defences in urban areas"

L121-122 'British and Irish Isles' – puzzled by this. If this is really important to you maybe say 'Britian and Ireland' as this makes sense to me.

> Thanks for this suggested simplification. To say "Britain and Ireland" would exclude Northern Ireland, strictly speaking. Therefore we have updated the text to read "the UK and Ireland".

L136 Is storyline H2 'informing future coastal adaptation planning' or 'more widely emissions and adaptation planning'. I think the goal here is much more to avoid this outcome with climate mitigation rather than prepare an adaptation plan.

> Please see responses to earlier review comments that address this point.

Line 157-160 Ports are probably incrementally upgrading already? Port design manuals already include (small) allowances for sea-level rise.

> Thanks for the comment. We have added the following text to the sentence on ports and added a citation to the Associated British Ports Climate Change Adaptation Report (https://www.abports.co.uk/media/q3lpq4mp/associated-british-ports-climate-change-adaptation-report-update-2021.pdf):

"... following port planning guidance and sector-specific climate change risk assessments[31] (Associated British Ports, 2021)."

In addition to the revisions requested by the reviewers, and in light of the recent paper by Seeger and Minderhoud (2026), we have clarified the datum used in the flood modelling in the Methods section as follows:

"The vertical datum is Ordnance Datum Newlyn in Great Britain and Ordnance Datum Belfast in Northern Ireland."
